# Culture Condition-Dependent Acylation Patterns of Trichothecenes in a T-2 Toxin-Producing Strain of *Fusarium sporotrichioides* NBRC 9955

**DOI:** 10.3390/ijms27021030

**Published:** 2026-01-20

**Authors:** Kazuyuki Maeda, Yuya Tanaka, Yuichi Nakajima, Kosuke Matsui, Yoshiaki Koizumi, Shuichi Ohsato, Naoko Takahashi-Ando, Makoto Kimura

**Affiliations:** 1 Graduate School of Bioagricultural Sciences, Nagoya University, Furo-cho, Chikusa-ku, Nagoya 464-8601, Aichi, Japan; painominoru24@yahoo.co.jp (Y.T.); ynakajima.gm@gmail.com (Y.N.); matsuik.toyo@gmail.com (K.M.); mkimura@agr.nagoya-u.ac.jp (M.K.); 2 Faculty of Agriculture, Meiji University, 1-1-1 Higashi-Mita, Tama-ku, Kawasaki 214-8571, Kanagawa, Japan; ohsato@meiji.ac.jp; 3 Graduate School of Science and Engineering, Toyo University, Kujirai 2100, Kawagoe 350-0815, Saitama, Japan; s46d02200015@toyo.jp (Y.K.); ando_n@toyo.jp (N.T.-A.)

**Keywords:** culture medium, *Fusarium sporotrichioides*, identification, side-chain modification, trichothecene

## Abstract

*Fusarium sporotrichioides* strain M-1-1, originally deposited as *Fusarium solani* IFO 9955 in 1974 and later moved to NBRC, is known for producing T-2 toxin. In addition to NRRL 3299, which was used in the United States to study T-2 toxin biosynthesis, NBRC 9955 has been extensively used for trichothecene research in Japan. To facilitate and accurately document studies on trichothecene biosynthesis using NBRC 9955, its phylogenetic classification and trichothecene metabolite profiles were determined. As anticipated, NBRC 9955 was classified as *F. sporotrichioides*, which exhibited a more distant phylogenetic relationship to other strains within the same species. Time-course TLC analyses demonstrated the accumulation of various deacetylated trichothecenes in yeast extract-rich liquid media during the late growth stages. Conversely, an increase in 3-*O*-acetylation of T-2 toxin was observed at late stages when cultivated in micronutrient-poor synthetic liquid medium. Northern blot analysis revealed that *Tri8* expression halted in cultures with the synthetic medium, which accounts for the growth stage-dependent 3-*O*-acetylation observed. On a brown rice flour solid medium, the fungal strain produced mixtures of T-2 toxin, neosolaniol, HT-2 toxin, and their 3-*O*-acetyl derivatives. These results highlight the risk of underestimating the levels of toxic trichothecene metabolites when using the standard contamination monitoring protocols.

## 1. Introduction

Initially documented as a human disease called alimentary toxic aleukia (ATA) in the Soviet Union, the toxicosis also emerged among cattle consuming moldy corn in the United States during the early 1960s [1]. A research team in Wisconsin commenced investigations to determine the causative agent of moldy-grain toxicosis, subsequently isolating several fungi from the corn. The most toxic and prevalent fungal isolate was identified as *Fusarium tricinctum*, with several strains capable of producing potent toxins in pure cultures [2]. From the most toxic strain, T-2, Bamburg et al. isolated a novel toxin and elucidated its structure as 4β,15-diacetoxy-8α-(3-methylbutyryloxy)-12,13-epoxytrichothec-9-en-3α-ol, now commonly referred to as T-2 toxin [3]. This historical strain, deposited as NRRL 3299 in a culture collection at the Agricultural Research Service (ARS), U.S. Department of Agriculture (USDA), was later recognized as *Fusarium sporotrichioides* and employed as a model strain for studying the chemistry and genetics of trichothecene biosynthesis [4,5,6,7,8,9,10,11,12,13,14,15,16,17,18,19,20].

Around the same time, cases of equine intoxication were occasionally observed in the Tokachi district of Hokkaido, located in the north of Japan [21]. From the bean hulls used as feed, several toxigenic fungi were isolated, among which strain M-1-1 was identified as producing the largest quantities of T-2 toxin and neosolaniol (NEO) [22]. These investigations were conducted by a research team led by Yoshio Ueno, who is recognized for establishing the current classification system of trichothecenes into four types based on the side-chain modification patterns; type A (a single bond at C-8), type B (ketone at C-8), type C (an epoxide at C-7,8), and type D (macrocyclic ring between C-4 and C-15) [23,24]. Initially described as *Fusarium solani* [21], strain M-1-1 was later re-classified as *Fusarium sporotrichioides* based on the distinctive characteristics of polyblastic conidiogenous cells or polyphialides [25]. The Japanese research group has extensively utilized this strain in their studies on toxicology and fermentation of type A trichothecenes [21,22,23,26,27,28,29,30,31,32,33,34,35,36,37,38,39,40,41,42,43,44]. However, its precise phylogenetic position has not been confirmed through molecular analysis. Additionally, there is a need for a deeper understanding of complex trichothecene metabolite profiles from both chemical and molecular biological viewpoints [22,26,27,31].

Despite its importance and extensive genetic investigations, there is a lack of understanding regarding the sequence diversity and transcriptional regulation of trichothecene biosynthesis genes (*Tri* genes). The known *Tri* genes of this strain include *Tri101*, encoding trichothecene 3-*O*-acetyltransferase [45] (AB014491 and BAA33771), *Tri6*, encoding a Cys_2_His_2_ zinc finger transcription factor [46] (AB017497 and BAA83724), and *Tri13*, encoding a cytochrome P450 monooxygenase [47] (AB088350 and BAC66094). Apart from *Tri101*, which lies outside the core trichothecene gene cluster and groups phylogenetically with isolates from the same geographic region [48], the *Tri6* and *Tri13* sequences of strain M-1-1 are unusually divergent from those of other *F. sporotrichioides* strains in the NCBI database. The orthologous counterparts of these genes in other *F. sporotrichioides* strains show deduced amino acid sequence identities of less than 94% (Supplementary Figure S1; as of December 2025), as determined by blastp search against non-redundant protein sequences (nr) and tblastn search against whole-genome shotgun (wgs) contigs. In contrast, *Tri6* and *Tri13* of the historical reference strain NRRL 3299 have orthologous genes from strains S17/1, S17/16, S18/43, and FB2 with nearly identical (98–100%) amino acid sequences (Supplementary Figure S1).

Without updated taxonomic data within the Sporotrichiella section, our research group has utilized strain M-1-1 (initially deposited as IFO 9955 in 1974 and later transferred to NBRC in 2002) as a T-2 toxin producer for biosynthetic investigations [45,46,47,49,50,51,52]. To accurately document research on this historical strain, we have conducted a genetic characterization of its attributes, elucidating its trichothecene profiles from a biosynthetic perspective.

## 2. Results and Discussion

### 2.1. Phylogenetic Confirmation of F. solani Deposited as NBRC 9955

**Figure 1:**
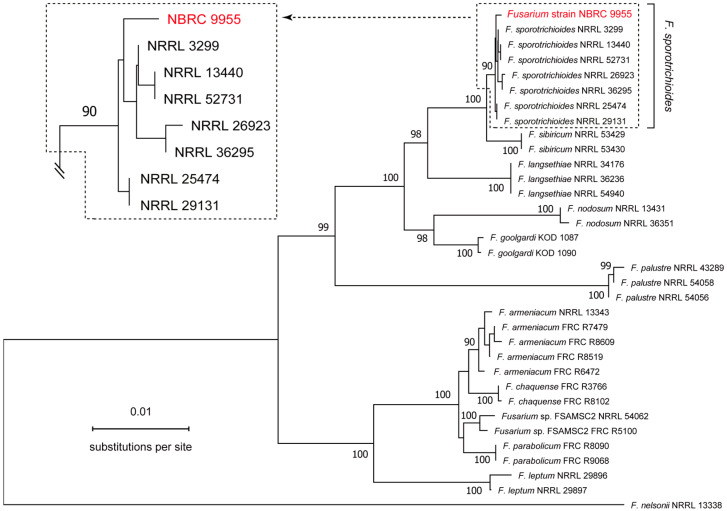
Molecular phylogenetic identification of *Fusarium* strain NBRC 9955. A neighbor-joining tree was constructed using the combined sequences of part of *RPB1*, *RPB2*, and *EF1α*. The analysis included NBRC 9955, along with 32 *Fusarium* species, with *F. nelsonii* NRRL 13338 serving as the outgroup [53]. Bootstrap values were calculated from 1000 replicates, with values ≥ 90% indicated at the branch nodes. The branch containing *F. sporotrichioides* strains was enlarged in the upper left corner. The DNA sequences used in these experiments are documented in Supplementary Table S1.

Upon confirming that the morphological characteristics of NBRC 9955 (Supplementary Figure S2) align with those documented by Ichinoe [25], we proceeded to examine its phylogenetic relationship with other *Fusarium* species. The phylogenetic analysis included 33 taxonomically verified species, comprising 7 strains of *F. sporotrichioides* (including two strains of *F. sporotrichioides* var. *minus*), 5 strains of *Fusarium armeniacum*, 3 strains of *Fusarium langsethiae*, 3 strains of *Fusarium palustre*, and 15 additional *Fusarium* species (Figure 1). The neighbor-joining tree, constructed using combined partial sequences of the largest and second largest subunit genes of the DNA-directed RNA polymerase II (*RPB1* and *RPB2*) [53] and the translation elongation factor-1 alpha (*EF1α*) gene [54,55,56], supported the classification of NBRC 9955 as *F. sporotrichioides*.

### 2.2. Sequencing of the Trichothecene Gene Cluster

To ascertain the distinctiveness of the cluster *Tri* gene sequences of strain NBRC 9955 among *F. sporotrichioides* strains, the sequences of the core gene cluster (*Tri5*-cluster) and the two-gene mini cluster (*Tri1*-cluster) were examined using Sanger sequencing (accessions LC785693 and LC785694). This study also included whole-genome shotgun sequencing to support molecular genetic research of NBRC 9955. The quality of this data set was evaluated by analyzing the sequences of several genes, as detailed in the legend of Supplementary Table S2.

**Figure 2:**
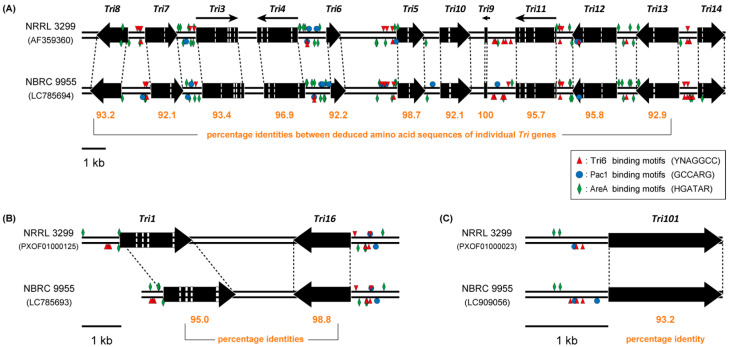
The trichothecene gene clusters of *F. sporotrichioides* strains NRRL 3299 and NBRC 9955. The sequences of the *Tri5* main gene cluster (accession: LC785694) and *Tri1*–*Tri16* mini gene cluster (accession: LC785693) of NBRC 9955 were determined by the Sanger method in this study. Panel (**A**) depicts the structure of the *Tri5* main gene cluster, while panel (**B**) presents the structure of the *Tri1*–*Tri16* mini gene cluster. Panel (**C**) shows the structure of the *Tri101* region (accession: LC909056). As the accession AB014491 [46] comprised only 73 bp of sequence upstream from the *Tri101* coding region, the promoter sequence was inferred using the whole-genome shotgun sequence of NBRC 9955 and subsequently confirmed by Sanger sequencing. The size and orientation of the *Tri* genes are indicated by black arrows, with predicted introns indicated as white lines. Within the predicted promoter region, located 1 kb upstream of the start codon, the consensus binding motifs for transcription factors implicated in trichothecene gene regulation are depicted as follows: a red triangle for Tri6 (YNAGGCC) [57], a blue circle for Pac1 (GCCARG) [58], and a green rhombus for AreA (HGATAR) [59]. In our investigation of self-cloning strains with mutations in the *Tri4*–*Tri6* bidirectional promoter region, analogous to the mutation analysis of the AreA-binding sequence [60], the Tri6p-binding motif mentioned above [57] was found to be critically important for trichothecene production. The numbers beneath the arrows represent the amino acid identity between the two strains.

The arrangement and orientation of the cluster genes were found to be similar to those of NRRL 3299 (Figure 2). However, the deduced amino acid sequence identities of the *Tri* genes between strains NBRC 9955 and NRRL 3299 were relatively lower, with the exception of *Tri5*, which exhibited greater than 98% identity. In addition to the previously mentioned *Tri6* and *Tri13* (Supplementary Figure S1), the *Tri* genes of strain NBRC 9955 were generally distantly related to those of strain NRRL 3299. Therefore, NBRC 9955 may have unique characteristics in the evolutionary process of the *Tri* genes among the known *F. sporotrichioides* strains.

### 2.3. Trichothecene Metabolites of Strain NBRC 9955 on Various Media

Previous investigations on the fermentation of this strain have identified the production of T-2 toxin, HT-2 toxin, 4,15-diacetoxyscirpenol (DAS), NEO, 15-deacetylneosolaniol (15-deNEO), and 8-acetoxy-15-deacetylneosolaniol (8-A-15-deNEO) when cultivated on complex media [21,22,26,31]. In contrast to *F. graminearum*, which typically requires the inducer molecule sucrose for trichothecene synthesis [61], *F. sporotrichioides* NBRC 9955 is capable of producing substantial quantities of T-2 toxin in liquid media without sucrose. To further elucidate the metabolite profiles of this strain, we monitored time-dependent changes in toxin profiles across various types of media. The ethyl acetate extracts from the cultures were visualized on thin-layer chromatography (TLC) through a color reaction with 4-(4-nitrobenzyl)pyridine/tetraethylenepentamine (NBP/TEPA) and subsequently confirmed via liquid chromatography-tandem mass spectrometry (LC-MS/MS) analysis.

**Figure 3:**
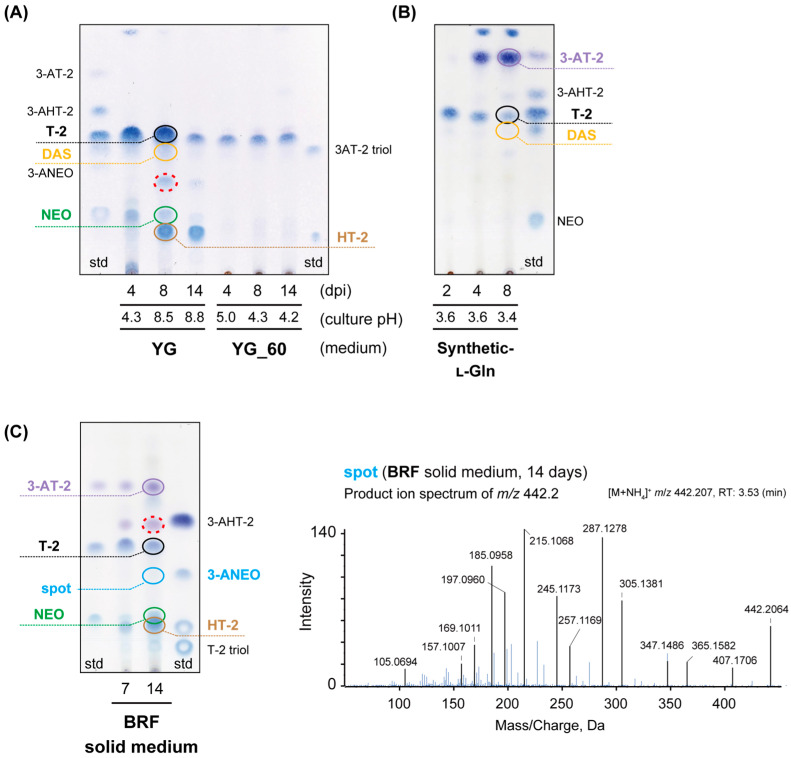
The trichothecene metabolites of *F. sporotrichioides* NBRC 9955 were extracted from cultures grown on various substrates, including YG and YG_60 liquid media (**A**), synthetic l-Gln liquid medium (**B**), and brown rice flour (BRF) solid medium (**C**). Ethyl acetate extracts derived from 1 mL of liquid or 0.25 g solid cultures were subjected to high-performance thin-layer chromatography (HPTLC). The spots, indicated by colored circles and red dotted circles, represent trichothecenes that were detected and undetected by liquid chromatography-tandem mass spectrometry (LC-MS/MS) in positive ion mode, respectively. The LC-MS/MS exhibited relatively low sensitivity for the detection of HT-2 toxin and 3-acetylHT-2 toxin (3-AHT-2), whereas it demonstrated satisfactory results for the neosolaniol (NEO) and 3-acetylneosolaniol (3-ANEO) detection. The right section of panel (**C**) illustrates the MS/MS fragmentation pattern of 3-ANEO, which is scarcely visible on a TLC plate. On TLC plates, blue spots that correspond to 3-AHT-2 were observed, yet no liquid chromatography–mass spectrometry (LC-MS) peaks were detected in positive ion mode. The MS/MS spectra of standard trichothecene samples analyzed under the same conditions are presented in Supplementary Figure S3.

In the nitrogen-rich YG liquid medium (Section 3.2) with reciprocal shaking, less acylated trichothecenes, NEO and HT-2 toxin were detected along with T-2 toxin (Figure 3A). Although barely visible on TLC, LC-MS/MS analysis confirmed the presence of DAS in the YG culture. Temporal changes in concentrations of trichothecene molecular species suggested that C-4 deacetylation of T-2 toxin led to the accumulation of HT-2 toxin in the YG medium (Figure 3A). Notably, the medium pH reached approximately 8 after 14 days of incubation, likely facilitating the hydrolysis of the ester linkage (Supplementary Figure S4). Additionally, an unidentified spot (marked by a red dotted circle) corresponding to an LC-MS peak of *m*/*z* 442.2 was detected on TLC. This peak is most likely [15-deacetylT-2 toxin (iso-HT-2 toxin) + NH_4_^+^] (*m*/*z* 442.2436), as its retention time and MS/MS fragmentation pattern differ from those of HT-2 toxin (Supplementary Figure S3B). Iso-HT-2 toxin was previously reported to be present in very low amounts in agar-medium cultures of *Fusarium* resembling *Fusarium poae* [62], but its structure has not yet been elucidated by nuclear magnetic resonance (NMR) analysis. When cultured in glucose-rich and nitrogen-poor YG_60 medium, the pH gradually decreased to 4, with the accumulation of T-2 toxin, albeit in smaller quantities than those obtained from the YG culture. Such trichothecene profiles were not observed for *F. sporotrichioides* strains MAFF 240356 and MAFF 240373, which produced lower quantities of T-2 toxin (Supplementary Figure S5).

Upon incubation of NBRC 9955 in a micronutrient-poor synthetic l-Gln medium with reciprocal shaking, a majority of T-2 toxin was converted into 3-acetylT-2 toxin (3-AT-2) after a 4-day incubation period (Figure 3B). A similar acetylation of T-2 toxin was observed during the later stages of culture when a micronutrient-poor synthetic medium with a mixture of amino acids (1.33 × NS) was employed (Supplementary Figure S6). This consistent modification of the side chain in the synthetic medium suggests a potential alteration in the expression of trichothecene (*Tri*) genes that modify the C-3 position, which is implicated in the self-protection mechanism of the trichothecene producer [11,45]. The accumulation of 3-AT-2 was most pronounced in the culture of NBRC 9955, which produces the largest amount of T-2 toxin in the synthetic l-Gln medium, whereas the degrees of C-3 acetylation were marginal in the cultures of MAFF 240356 and MAFF 240373 (Supplementary Figure S4).

NBRC 9955 was also cultivated on a brown rice flour (BRF) solid medium, and the metabolites were extracted using ethyl acetate. TLC combined with LC-MS/MS analyses revealed the presence of mixtures of various type A trichothecenes (Figure 3C), including T-2 toxin, 3-AT-2, NEO, 3-acetylneosolaniol (3-ANEO), and HT-2 toxin. Although the presence of 3-acetyl HT-2 toxin (3-AHT-2) was not confirmed by LC-MS/MS, TLC analysis revealed blue spots with an *R_f_* value identical to that of 3-AHT-2 on TLC (denoted by a red dotted circle). The low T-2 toxin-producing strains, MAFF 240356 and MAFF 240373, did not produce 3-*O*-acetylated trichothecenes on BRF medium (Supplementary Figure S7). These findings suggest that the large amounts of highly toxic trichothecenes produced by NBRC 9955, particularly T-2 toxin and NEO, require detoxification through 3-*O*-acetylation [63] to mitigate their toxicity under the culture conditions.

### 2.4. Altered Expression of the C-3 Deacetylase Gene Accounts for the Accumulation of 3-Acetyl Trichothecenes

**Figure 4:**
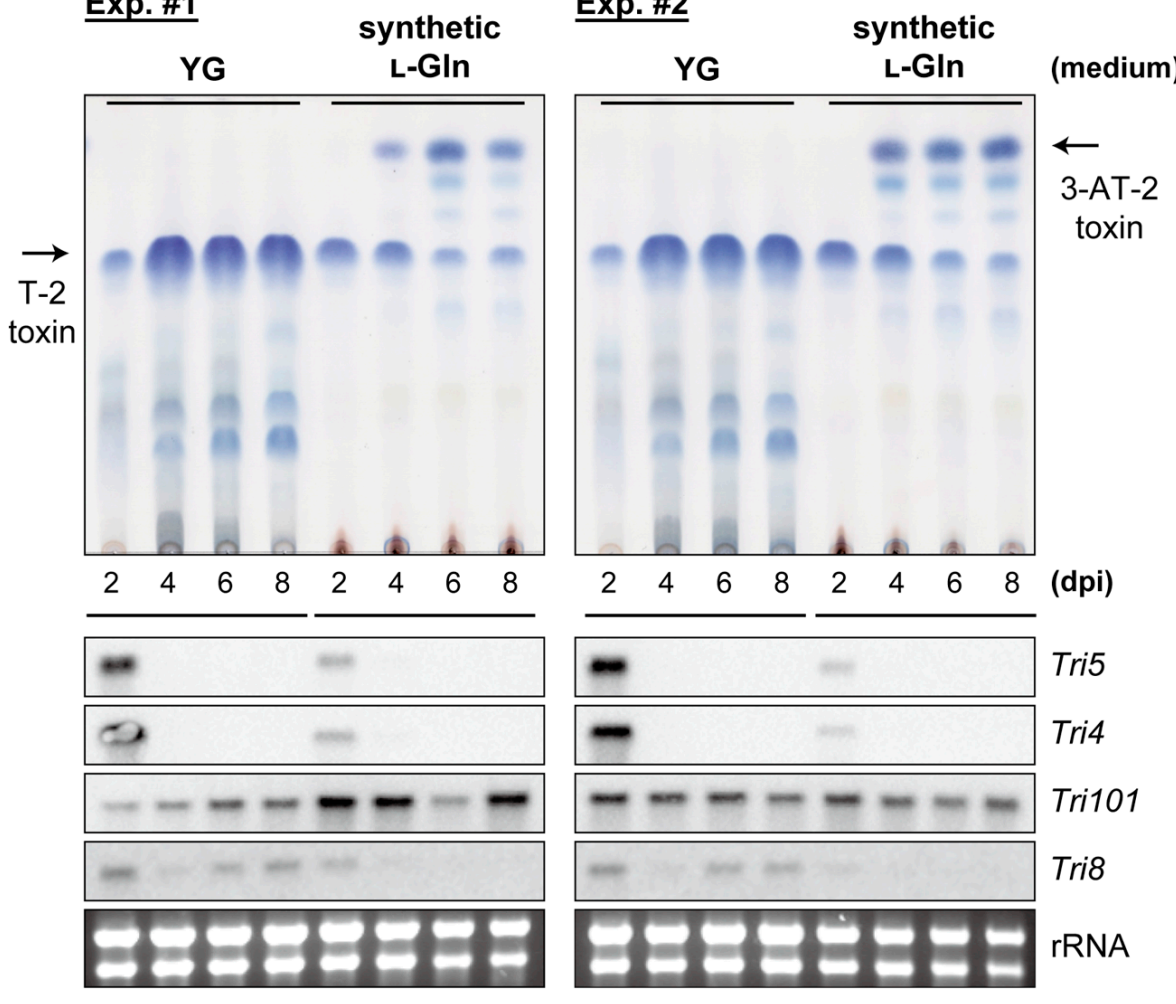
*Tri* gene expressions in strain NBRC 9955 cultured in YG and synthetic l-Gln liquid media. The upper panel displays TLC of trichothecenes extracted from the liquid culture. Analysis of *Tri* gene expression in liquid cultures by northern blotting is shown in the lower panel. Duplicate experiments were performed using fungal cultures prepared at different times.

To elucidate the molecular mechanism underlying the accumulation of 3-AT-2 during the late stages of culture in synthetic l-Gln medium, the temporal expression of *Tri* genes in NBRC 9955 was analyzed. The presence or absence of an acetyl group at C-3 is determined by the competing activities of C-3 acetylase and deacetylase, encoded by *Tri101* and *Tri8*, respectively [13,45]. As these two genes are not situated within the core region of the trichothecene gene cluster [64], the transcription analysis also included the tightly regulated pathway enzyme genes, *Tri4* and *Tri5*, for comparative purposes [65]. As shown in Figure 4, northern blot analyses revealed that the expression of *Tri101* remained consistently high, irrespective of the culture medium type, even after the transcription of *Tri4* and *Tri5* had ceased. However, in the synthetic l-Gln medium, transcription of *Tri8* ceased at an early stage when *Tri4* and *Tri5* mRNA were no longer detectable, as opposed to the YG culture, where *Tri8* continued to be expressed. Therefore, the accumulation of 3-acetyltrichothecenes at a later stage of the NBRC 9955 culture is likely due to the altered expression of *Tri8* under the culture conditions.

### 2.5. Proposed Mechanism Generating the Side-Chain Diversity of Trichothecenes on Different Media

**Figure 5:**
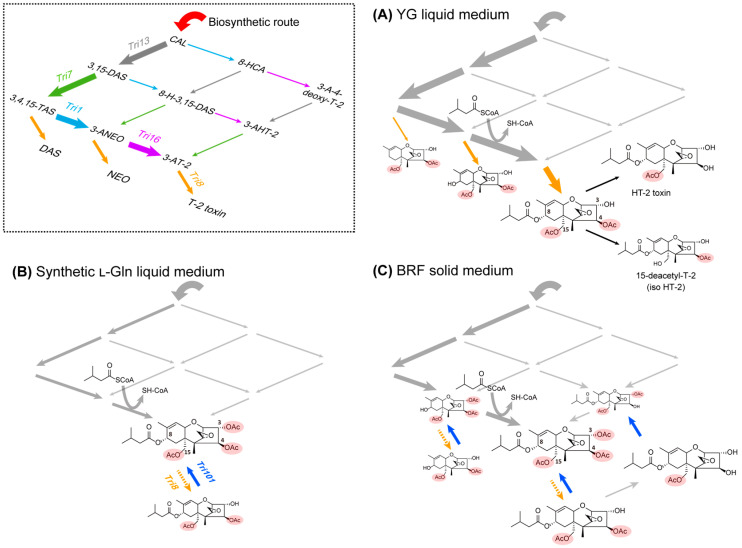
Proposed biosynthetic routes to various trichothecene compounds that accumulated in YG liquid (**A**), synthetic l-Gln liquid (**B**), and BRF solid (**C**) cultures of *F. sporotrichioides* strain NBRC 9955. In YG liquid culture, iso-HT-2 toxin is proposed to have originated through non-specific hydrolysis of an ester bond in T-2 toxin, as suggested by the presence of a peak with the same *m*/*z* value as the HT-2 toxin in LC-MS/MS analysis (Supplementary Figure S3B). A small amount of 3-AHT-2 was detected on BRF solid culture, but only through TLC (Figure 3C), likely due to the relatively low sensitivity of LC-MS/MS in positive ion mode for detecting HT-2 toxin and related compounds.

As described above, the trichothecene profiles in cultures of *F. sporotrichioides* NBRC 9955 are markedly affected by the type of medium used. In examining these alterations in trichothecene profiles, the temporal changes in the concentration of each trichothecene molecular species (Figure 3) provide insights into the direction of metabolic flow within the T-2 toxin biosynthetic grid. For example, when a highly trichothecene-inducing YG medium for NBRC 9955 was used, the isovalerate supply appeared inadequate to enable complete esterification at C-8 (Figure 5A), resulting in the accumulation of NEO to a certain degree (Figure 3A). Similarly, the increased medium pH during the later cultivation period may explain the production of HT-2 toxin and putative iso-HT-2 toxin from T-2 toxin in the YG culture (Figure 3A). Such deacetylated trichothecenes were not detected in the YG_60 and synthetic l-Gln cultures (Figure 3A,B), where toxin productivity and medium pH were lower than those in the YG culture (Figure 5B,C). NEO was also observed during the earlier cultivation period on solid culture with the BRF medium (Figure 3C), suggesting the operation of a shunt pathway similar to that observed in the YG culture.

Notably, the use of synthetic l-Gln medium resulted in the accumulation of 3-AT-2 (Figure 3C), a phenomenon attributable to the transcriptional downregulation of the *Tri8* deacetylase gene (Figure 4). Given that the highly toxic T-2 toxin, once secreted into the medium, can re-enter fungal cells under specific culture conditions, the promotion of 3-*O*-acetylation may represent a strategic adaptation for the survival of the producing organism. Similarly, time-dependent increases in 3-AT-2 levels on BRF solid medium suggest the presence of a similar mechanism in the highly toxin-producing strain NBRC 9955 (Figure 3C). Moreover, although the production is less efficient than that by NBRC 9955, strain NRRL 3287 of *F. sporotrichioides* (formerly described as *Fusarium poae*) has been documented to produce 3-AT-2 in a Richards solution medium [66,67]. Consequently, the patterns of trichothecene acylation likely reflect the biotic and abiotic conditions surrounding the T-2 toxin-producing *Fusarium* strains.

## 3. Materials and Methods

### 3.1. Strains

T-2 toxin-producing *Fusarium* strains used in this study were sourced from the National Institute of Technology and Evaluation (NITE)-Biological Resource Center (NBRC, Kisaradzu, Japan) and the National Institute of Agrobiological Sciences (NIAS) Genebank (Tsukuba, Japan), under the Ministry of Agriculture, Forestry and Fisheries (MAFF). These strains included *F. sporotrichioides* NBRC 9955 (deposited as *F. solani*), as well as MAFF 240356 and MAFF 240373 (designated as approved strains of *F. sporotrichioides* in the NIAS Genebank). A *Tri7* gene disruptant (Δ*tri7::hph::tk*) of NBRC 9955 was generated in this study, as described in the legend of Supplementary Figures S8 and S9, and used for the production of HT-2 toxin.

### 3.2. Media and Reagents

Fungal strains were maintained on V8-juice agar media (20% Campbell’s V8-juice, 0.3% CaCO_3_, 1.5% agar), with or without the addition of hygromycin B (300 µg/mL), at 25 °C in the dark. Conidia were induced on CMC liquid medium (1.5% CMC sodium salt, 0.1% NH_4_NO_3_, 0.1% KH_2_PO_4_, 0.1% yeast extract, 0.05% MgSO_4_·7H_2_O) by reciprocal shaking at 125 strokes/min and 25 °C. The trichothecene profiles of fungal strains were examined by using YG liquid (0.5% yeast extract, 2% glucose), YS liquid (0.5% yeast extract, 2% sucrose), YG_60 liquid (0.1% yeast extract, 6% glucose), YS_60 liquid (0.1% yeast extract, 6% sucrose), synthetic l-Gln liquid (5 mM l-glutamine as the nitrogen source; Supplementary Table S3), synthetic NaNO_3_ liquid (5 mM NaNO_3_ as the nitrogen source; Supplementary Table S3), and BRF solid medium (Supplementary Table S3). BD Bacto™ yeast extract (Lot No. 1186275 and No. 6295747), hygromycin B, NBP, TEPA, and other chemicals were purchased from FUJIFILM Wako Pure Chemical Corporation (Osaka, Japan). High-performance TLC (HPTLC) plates (Glass HPTLC plate, silica gel 60 coated with fluorescent indicator F_254_), TLC plates (Glass TLC plate, silica gel coated with F_254_), and hyper-grade acetonitrile for liquid chromatography–mass spectrometry (LC-MS) were obtained from Merck KGaA (Darmstadt, Germany).

### 3.3. Molecular Phylogenetic Analysis

Partial sequences of *RPB1*, *RPB2*, and *EF1α* of relevant *Fusarium* species were amplified by PCR and sequenced using the Sanger method, employing the primers listed in Supplementary Table S4. The DNA sequences obtained, along with available reference sequences from previous studies (Supplementary Table S1), were used for molecular phylogenetic analysis. The combined sequences of *RPB1*, *RPB2*, and *EF1α* were aligned using the Clustal W method provided by MEGA 11 software [68]. A neighbor-joining tree was constructed, and bootstrap values (1000 replicates) for subtrees were calculated based on Kimura’s two-parameter method for multiple alignment data.

### 3.4. Preparation of Genomic DNA and Whole Genome Sequencing

For whole genome sequencing, genomic DNA of strain NBRC 9955 was extracted by using NucleoBond^®^ HMW DNA (Takara Bio Inc., Kusatsu, Japan). The sequencing library was constructed and sequenced using NovaSeq 6000 with the S4 Reagent Kit (2 × 150 bp paired-end reads, Illumina Inc., San Diego, CA, USA) by Eurofins Genomics K.K. Ltd. (Tokyo, Japan). The total number of reads for the strain was 38,881,734. The percentage of reads with a quality score was 94.47%. The reads were analyzed by GENETYX-NGS (version 4.1.1, GENETYX Corporation, Tokyo, Japan). Nucleotide sequence data are available in the short sequence archive under the NCBI accession DRR787368.

### 3.5. Culture Conditions for Toxin Production

Thirty microliters of conidia, adjusted to a concentration of 1 × 10^7^ conidia/mL, were inoculated onto 30 mL of each medium: YG, YS, YG_60, YS_60, synthetic l-Gln, and synthetic NaNO_3_ medium, in a 100-mL Erlenmeyer flask. The liquid cultures were maintained at 28 °C with reciprocal shaking at 125 rpm. Samples were extracted from 3 mL culture filtrates on appropriate days using an equal volume of ethyl acetate. For the solid culture, 5 × 10^5^ conidia (500 µL) were uniformly mixed with 5 g of autoclaved BRF solid medium, prepared in a 100-mL culture bottle (2-085-02, AS ONE Co., Osaka, Japan) with an aluminum cap. Following incubation at 25 °C for 14 days with daily stirring, the solid culture was extracted with 20 mL of 84% (*v*/*v*) acetonitrile and filtered through filter paper No.1 (Toyo Roshi Kaisha, Ltd., Tokyo, Japan). The fungal metabolites were dried under a nitrogen stream and extracted twice with ethyl acetate.

### 3.6. Trichothecene Standards and Analytical Methods

T-2 toxin, T-2 triol, DAS, NEO, and T-2 tetraol were purchased from Wako Pure Chemical Industries and Sigma-Aldrich (St. Louis, MO, USA). Scirpenol was prepared by treating DAS with ammonium solution as described previously [52]. HT-2 toxin was purified from the *tri7* disruptant. 3-AT-2 toxin, 3-AHT-2 toxin, 3-acetylT-2 triol (3AT-2 triol), 3-acetoxyscirpenol, 15-acetoxyscirpenol, 3,15-diacetoxyscirpenol, 3-ANEO, 3-acetylT-2 tetraol, 15-acetylT-2 tetraol, and 3,15-diacetylT-2 tetraol were prepared by enzymatic 3-*O*-acetylation (by rTRI101) and/or 15-*O*-acetylation (by rTRI3) of available trichothecene standards [69].

The samples were applied to (HP)TLC plates and developed with ethyl acetate-toluene in a 3:1 ratio. Trichothecenes were visualized as blue-purple spots through a color reaction with NBP/TEPA [70]. Additionally, trichothecenes were analyzed via LC-MS/MS in positive ion mode. The samples were separated using the Eksigent ekspert™ ultraLC 100-XL system (Eksigent Technologies, Dublin, CA, USA) connected to an AB SCIEX Triple TOF 4600 System (Framingham, MA, USA) equipped with a C18 reverse phase column (PEGASIL ODS SP100-3; 2φ × 200 mm, Senshu Scientific, Tokyo, Japan), as previously described [52].

### 3.7. Northern Blot Analysis of Tri Genes

Five µg of total RNA samples were subjected to separation on agarose gels containing formaldehyde following heat denaturation and subsequently transferred to a positively charged nylon membrane (Roche Diagnostics GmbH, Mannheim, Germany). Probes were generated by labeling DNA fragments with digoxigenin (DIG) using the PCR DIG Probe Synthesis Kit (Roche). The amplified cDNA fragment for *Tri5*, *Tri4*, *Tri101*, or *Tri8* was cloned into pGEM^®^-T Easy vector (Promega Co., Madison, WI, USA) and used as a template for the probe synthesis. The processes of hybridization, washing, and detection were conducted in accordance with the manufacturer’s protocol. The primers listed in Supplementary Table S4 were employed for the preparation of the DNA probes.

### 3.8. Accession Numbers

The nucleotide sequences of the trichothecene gene clusters were deposited at DDBJ/EMBL/Genbank (DNA Databank of Japan/European Molecular Biology Laboratory/Genbank) under accession numbers LC785693 and LC785694. Raw sequencing data of whole-genome shotgun contigs were deposited in the DDBJ/SRA database under BioProject number PRJDB35982. The dataset can be accessed in the short sequence archive (SRA) with accession number DRR787368.

## 4. Conclusions

We have identified NBRC 9955 as *F. sporotrichioides* based on molecular taxonomy and have characterized the unique features of this historical strain, emphasizing the chemistry and biology of trichothecene biosynthesis. It is noteworthy that strain NBRC 9955 produced various type A trichothecenes, including those not produced by other *F. sporotrichioides* strains, depending on the media used. This variability raises the risk of underestimating trichothecene contamination when employing standard analytical protocols for sample screening.

## Data Availability

The original contributions presented in this study are included in the article/Supplementary Materials. Further inquiries can be directed to the corresponding author.

## Supplementary Materials

The following are available online at https://www.mdpi.com/article/10.3390/ijms27021030/s1. Refs. [53,54,55,56,60,71,72,73,74,75,76,77,78,79,80,81,82,83,84,85,86,87,88,89] are cited in Supplementary Materials file.

## Abbreviations

The following abbreviations are used in this manuscript:

15-deNEO	15-Deacetylneosolaniol
3,15-DAS	3,15-Diacetoxyscirpenol
3,4,15-TAS	3,4,15-Triacetoxyscirpenol
3-A-4-deoxy T-2	3-Acetyl-4-deoxy T-2 toxin
3-AHT-2	3-AcetylHT-2 toxin
3-ANEO	3-Acetylneosolaniol
3-AT-2	3-AcetylT-2 toxin
4,15-DAS	4,15-Diacetoxyscirpenol
8-A-15-deNEO	8-Acetoxy-15-deacetylneosolaniol
8-HCA	8-Hydoxycalonectrin
8-H-3,15-DAS	8-Hydroxy-3,15-diacetoxyscirpenol
ATCC	American Type Culture Collection
BRF	Brown rice flour
CAL	Calonectrin
CMC	Carboxymethyl cellulose
DDBJ	DNA Databank of Japan
dpi	Days post inoculation
EMBL	European Molecular Biology Laboratory
hph	Hygromycin B phosphotransferase gene
HPTLC	High performance thin layer chromatography
IFO	Institute for Fermentation, Osaka
JCM	Japan Collection of Microorganisms
LC-MS	Liquid chromatography–mass spectrometry
LC-MS/MS	Liquid chromatography-tandem mass spectrometry
MAFF	Ministry of Agriculture, Forestry and Fisheries
NBP	4-(4-Nitrobenzyl)pyridine
NBRC	National Institute of Technology and Evaluation-Biological Resource Center
NEO	Neosolaniol
NIAS	National Institute of Agrobiological Sciences
NMR	Nuclear magnetic resonance
NRRL	Northern Regional Research Laboratory
Rf	Retention factor
RT	Retention time
std	Standard
TEPA	Tetraethylene pentamine
TLC	Thin layer chromatography
wgs	Whole genome shotgun
