# Supplementary material for: Culture Condition-Dependent Acylation Patterns of Trichothecenes in a T-2 Toxin-Producing Strain of Fusarium sporotrichioides NBRC 9955
Source: Int J Mol Sci. 2026 Jan 20;27(2):1030. doi: 10.3390/ijms27021030 (PMC12841802; doi:10.3390/ijms27021030)
Supplement: Supplementary file 1 [file ijms-27-01030-s001.zip › ijms-4027042-supplementary.pdf]

Supplementary Materials

Culture Condition-Dependent Acylation Patterns of Trichothecenes in a T-2 Toxin-Producing Strain of *Fusarium sporotrichioides* NBRC 9955

Kazuyuki Maeda [1,2,*], Yuya Tanaka [1], Yuichi Nakajima [1], Kosuke Matsui [1,3], Yoshiaki Koizumi [3], Shuichi Ohsato [2], Naoko Takahashi-Ando [3] and Makoto Kimura [1]

[1] Graduate School of Bioagricultural Sciences, Nagoya University, Furo-cho, Chikusa-ku, Nagoya 464-8601, Aichi, Japan
[2] Faculty of Agriculture, Meiji University, 1-1-1 Higashi-Mita, Tama-ku, Kawasaki 214-8571, Kanagawa, Japan
[3] Graduate School of Science and Engineering, Toyo University, Kujirai 2100, Kawagoe 350-0815, Saitama, Japan
* Correspondence: kmaeda@agr.nagoya-u.ac.jp; Tel.: +81-52-789-5744

Tri6 (Q00856)

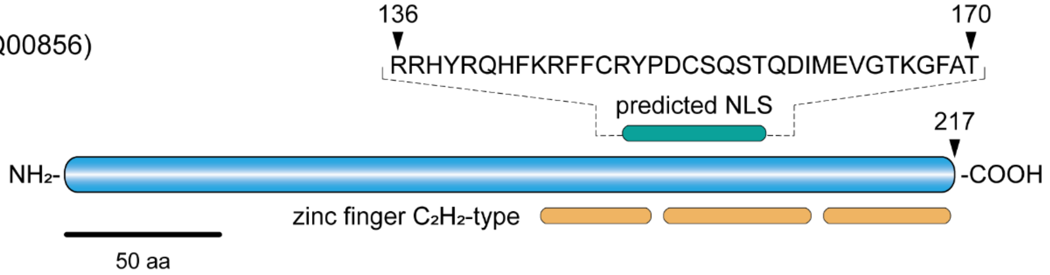

Tri6	NBRC 9955 BAA83724	S17/1 JAHKNT010000027	S17/16 JAHKNS010000002	S18/43 JAHKNR010000001	FB2 JAYJJV010000075	NRRL 3299 Q00856
NBRC 9955 218 aa	100%	93.6%	93.6%	92.2%	93.1%	92.2%
S17/1 217 aa		100%	100%	98.6%	99.5%	98.6%
S17/16 217 aa			100%	98.6%	99.5%	98.6%
S18/43 217 aa				100%	98.2%	100%
FB2 217 aa					100%	98.2%
NRRL 3299 217 aa						100%

90 – 100% amino acid identity

Tri13 (Q9C1I4)

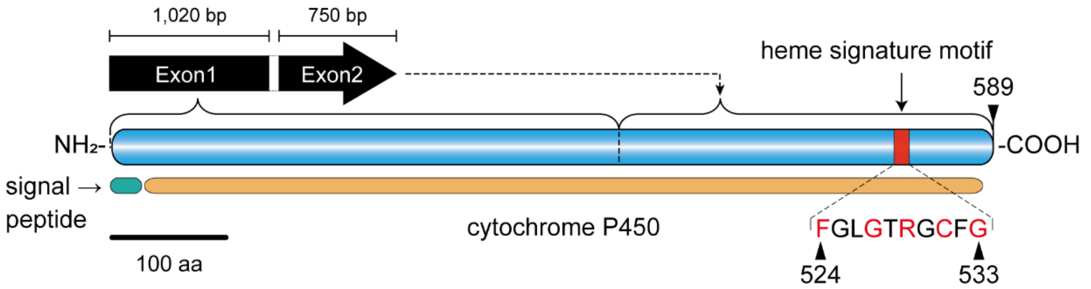

Tri13	NBRC 9955 BAC66094	S17/1 JAHKNT010000027	S17/16 JAHKNS010000002	S18/43 JAHKNR010000001	FB2 JAYJJV010000075	NRRL 3299 Q9C1I4
NBRC 9955 340+249 aa	100%	93.4%	92.9%	93.2%	93.2%	92.9%
S17/1 340+249 aa		100%	99.0%	99.5%	99.3%	99.0%
S17/16 340+249 aa			100%	99.2%	98.8%	100%
S18/43 340+249 aa				100%	98.8%	99.2%
FB2 340+249 aa					100%	98.8%
NRRL 3299 340+249 aa						100%

90 – 100% amino acid identity

Supplementary Figure S1. Divergence of Tri6 and Tri13 protein sequences of NBRC 9955 among those of *F. sporotrichioides* strains available in the NCBI database. A list of

Tri6 and *Tri13* orthologues, including *F. sporotrichioides* strains NRRL 3299, S17/1, S17/16, S18/43, and FB2, was compiled through a blastp search against non-redundant protein sequences (nr) and tblastn search against whole-genome shotgun (wgs) sequence contigs of *F. sporotrichioides*. The sequence identities between proteins are depicted in the figure, with a higher percentage identity indicated by a more intense background color.

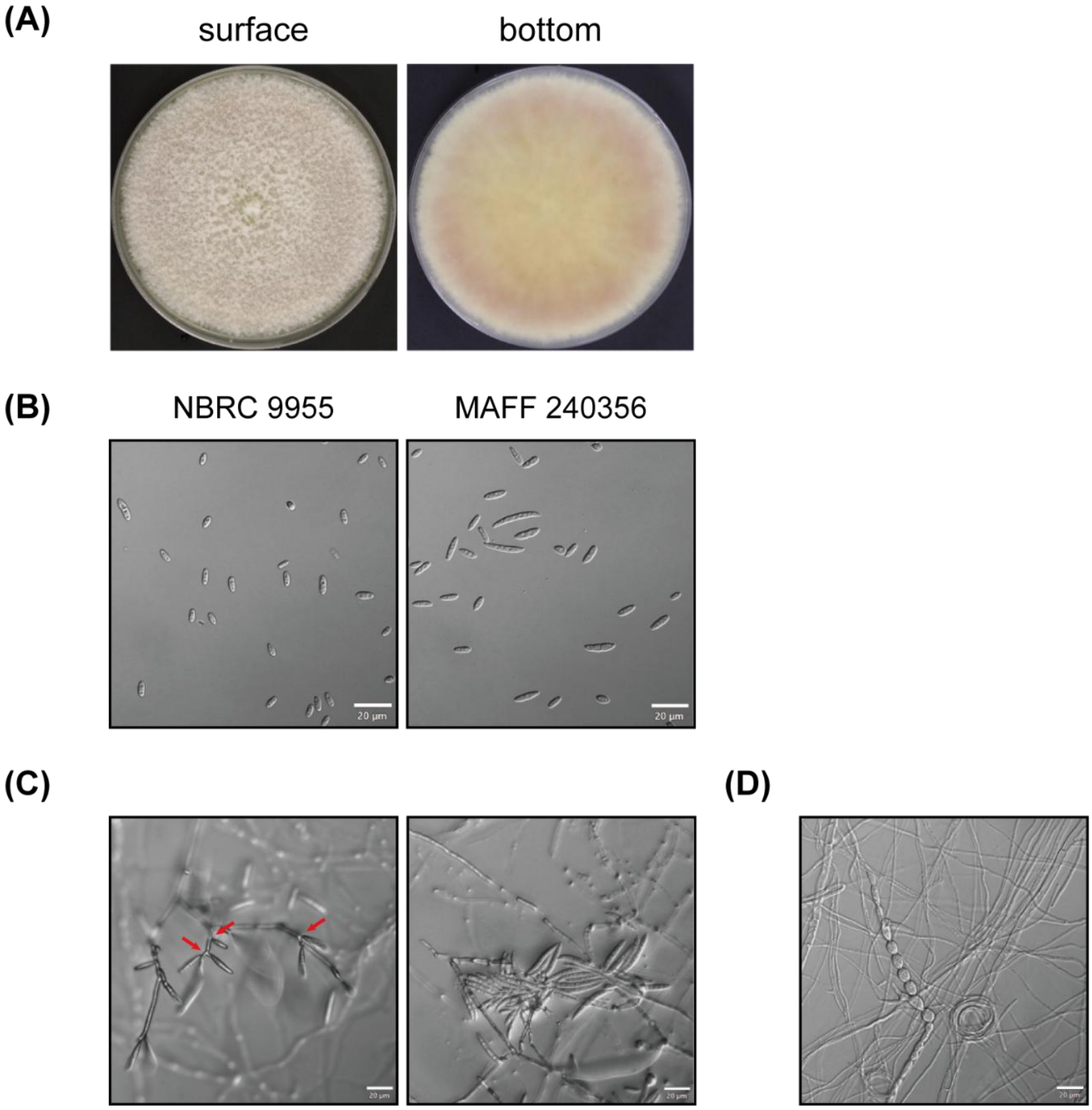

Supplementary Figure S2. Morphology and pigmentation of *Fusarium* strain NBRC 9955 in comparison to the reference strain, *F. sporotrichioides* MAFF 240356. (**A**) Surface (left) and bottom (right) views of the gigantic colony. Strain NBRC 9955 was cultivated on potato-dextrose agar plates at 25°C for 14 days. (**B**) Morphology of conidia of NBRC 9955 (left) and MAFF 240356 (right). The scale bars represent 20 μm. Conidia were harvested following a 3-day incubation period in carboxymethyl cellulose (CMC) liquid medium. In CMC liquid medium, strain NBRC 9955 did not produce macroconidia. The conidia, exhibiting multiple or no septa, were obovoid (egg-shaped) or pyriform (pear-shaped) in shape, which are somewhat broader than those of reference *F.*

sporotrichioides strains [1]. (**C**) Microconidia (left) and macroconidia (right) of NBRC 9955 formed on sterilized filter paper placed on a spezieller nährstoffarmer agar (SNA; special low-nutrient agar) under BLB (black light blue) light illumination [2]. The presence of polyphialides and polyblastic conidiogenous cells (denoted by red arrows) is characteristic of *F. sporotrichioides*, distinguishing it from *F. poae* and *F. langthesiae* [3, 4]. Strain NBRC 9955 formed microconidia, and very rarely, macroconidia, resembling those of typical *F. sporotrichioides* species. (**D**) Chlamydospores of NBRC 9955 formed on carnation leaf agar under BLB light illumination. Sporodochia was not observed on all the media used for the investigation. The scale bar measures 20 μm.

(A-1) T-2 toxin $[C_{24}H_{34}O_9 + H]^+$ *m/z*: 467.228, RT: 4.35 min

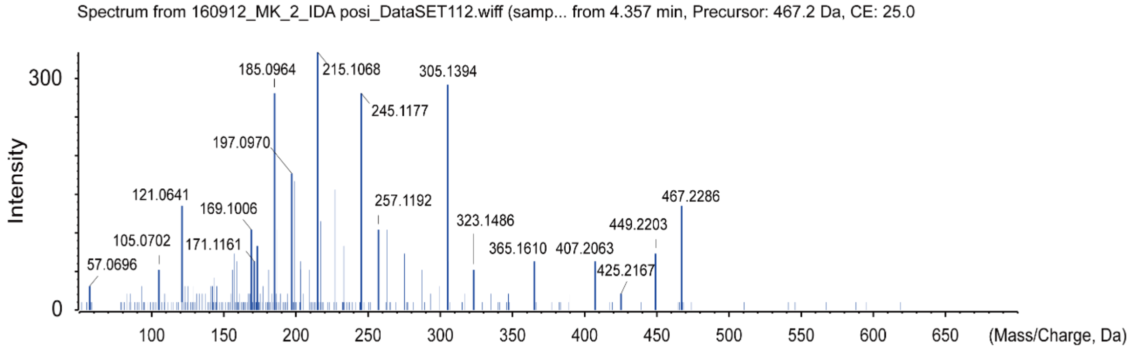

(A-2) T-2 toxin $[C_{24}H_{34}O_9 + NH_4]^+$ *m/z*: 484.254, RT: 4.35 min

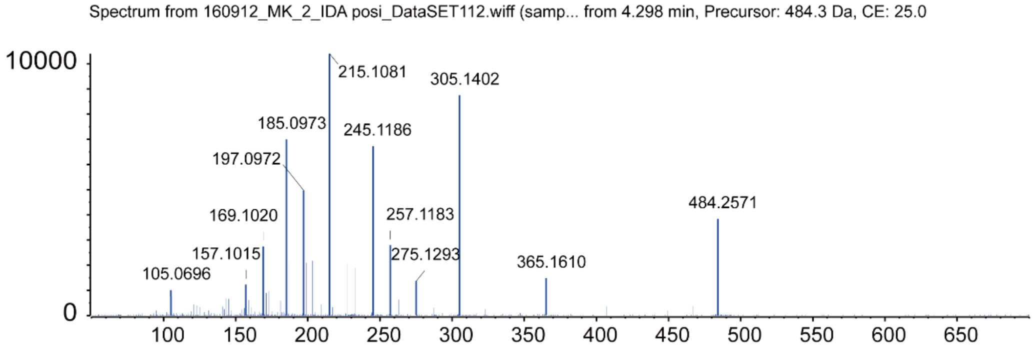

(B-1) HT-2 toxin $[C_{22}H_{32}O_8 + H]^+$ *m/z*: 425.217, RT: 3.78 min

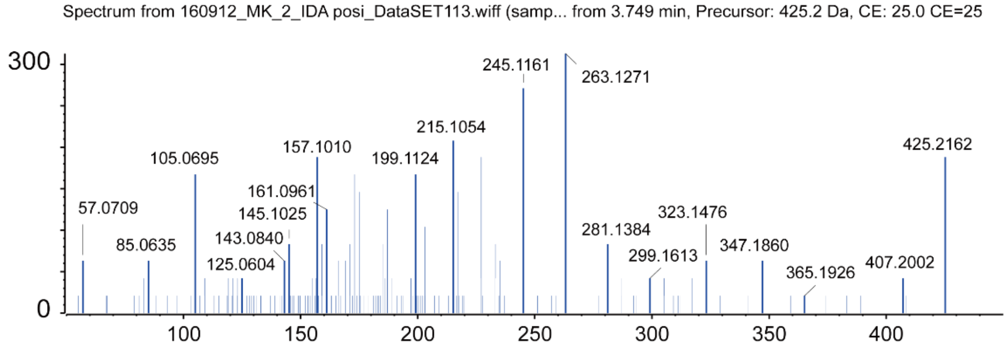

(B-2) HT-2 toxin $[C_{22}H_{32}O_8 + NH_4]^+$ *m/z*: 442.244, RT: 3.72 min

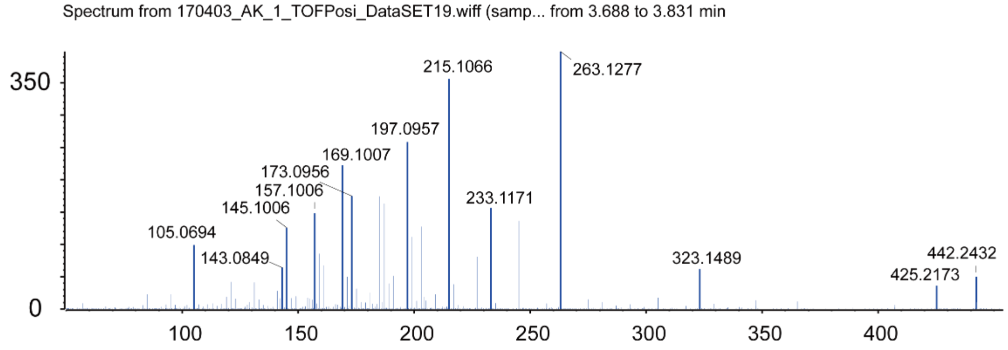

(C-1) 3-acetyl-T-2 toxin [$C_{26}H_{36}O_{10}$ + H]$^+$ *m/z*: 509.238, RT: 4.94 min

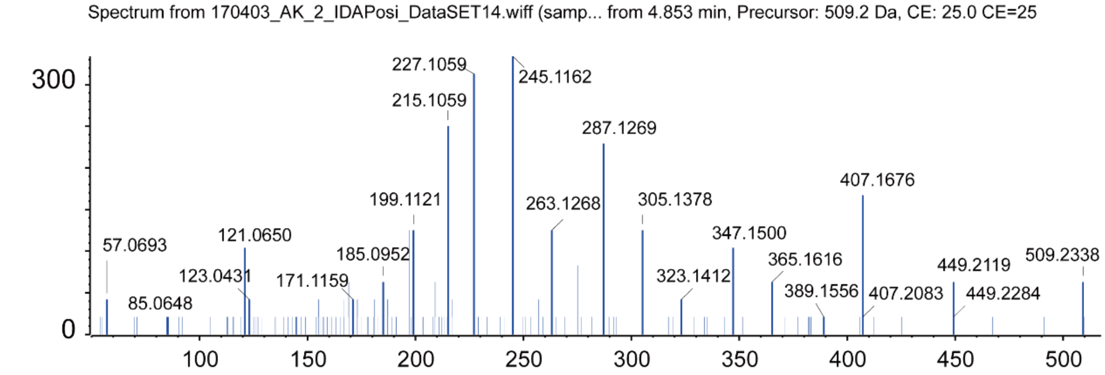

(C-2) 3-acetyl-T-2 toxin [$C_{26}H_{36}O_{10}$ + NH_4]$^+$ *m/z*: 526.265, RT: 4.99 min

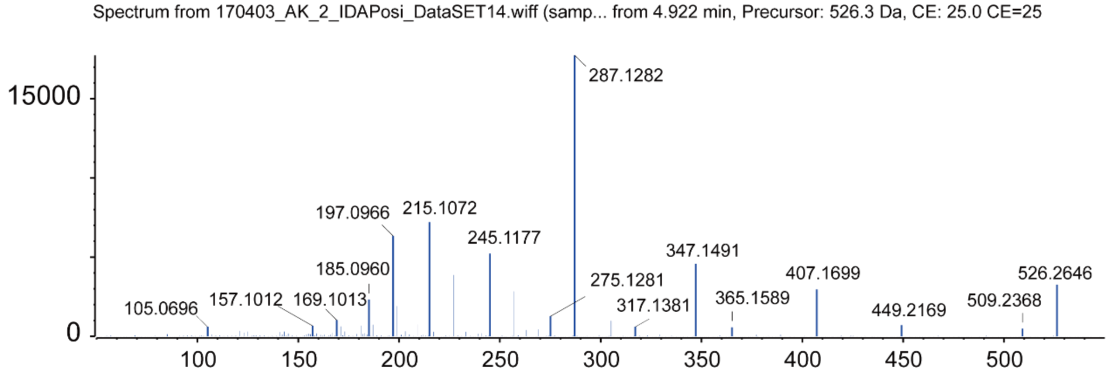

(D-1) T-2 triol [$C_{20}H_{30}O_7$ + H]$^+$ *m/z*: 383.206, RT: 3.39 min

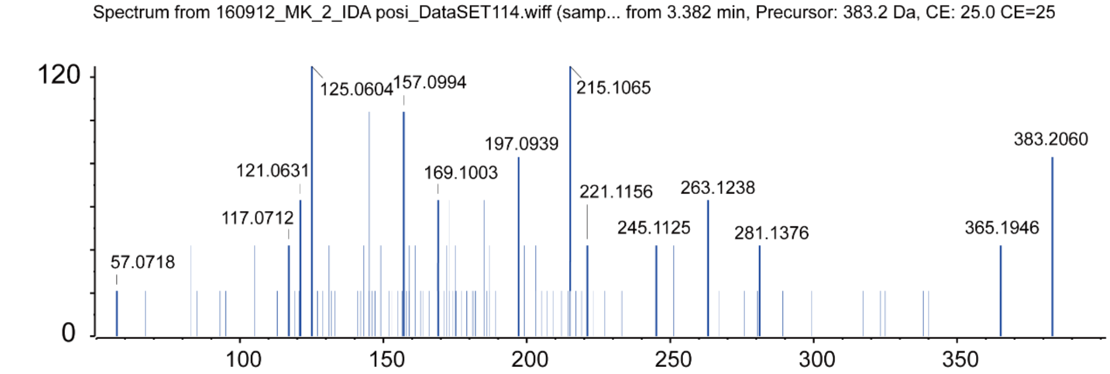

(D-2) T-2 triol [$C_{20}H_{30}O_7$ + NH_4]$^+$ *m/z*: 400.233, RT: 3.47 min

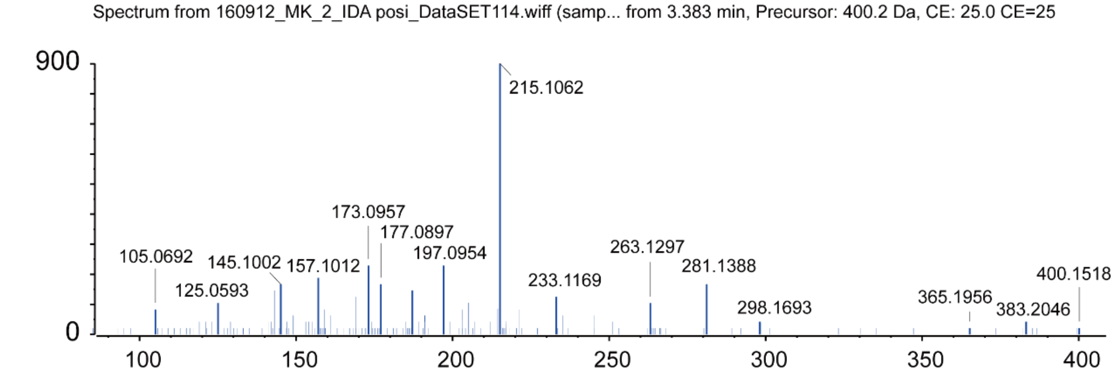

(E-1) 3-acetyl-T-2 triol [$C_{22}H_{32}O_8$ + H]$^+$ *m/z*: 425.217, RT: 4.02 min

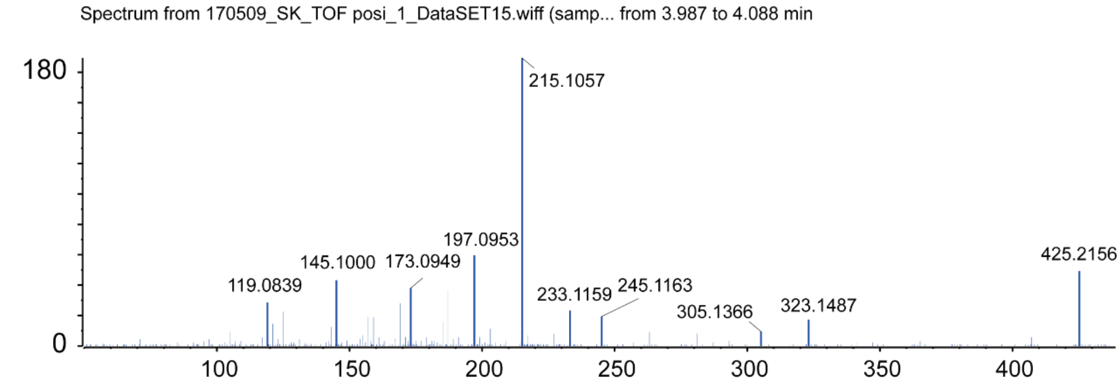

(E-2) 3-acetyl-T-2 triol [$C_{22}H_{32}O_8$ + NH_4]$^+$ *m/z*: 442.244, RT: 4.03 min

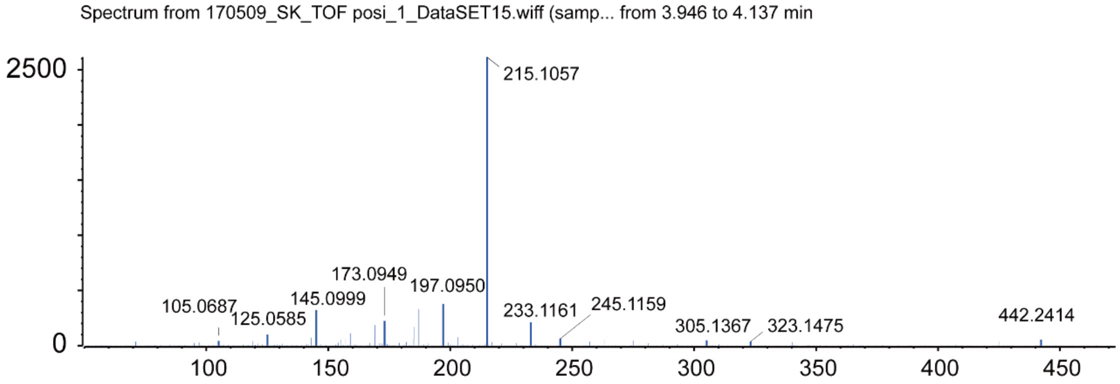

(F) T-2 tetraol [$C_{15}H_{22}O_6$ + H]$^+$ *m/z*: 299.149, RT: 1.90 min

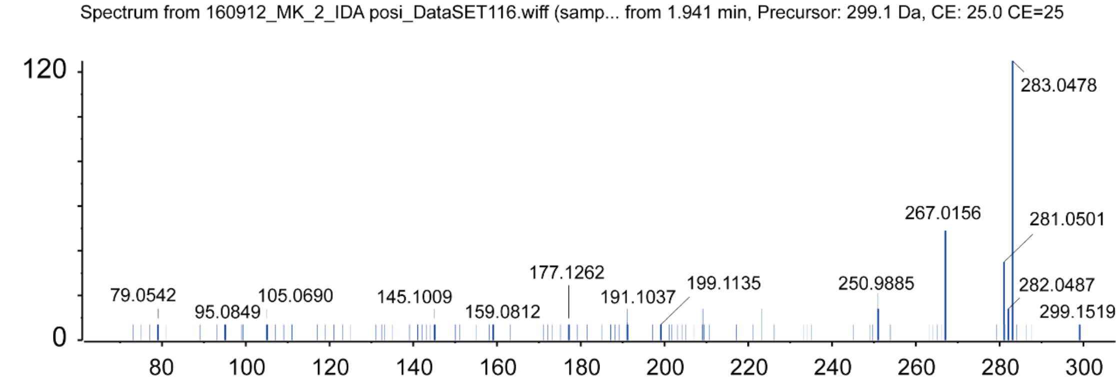

(G) 3-acetyl-T-2 tetraol $[C_{17}H_{24}O_7 + NH_4]^+$ *m/z*: 358.186, RT: 2.62 min

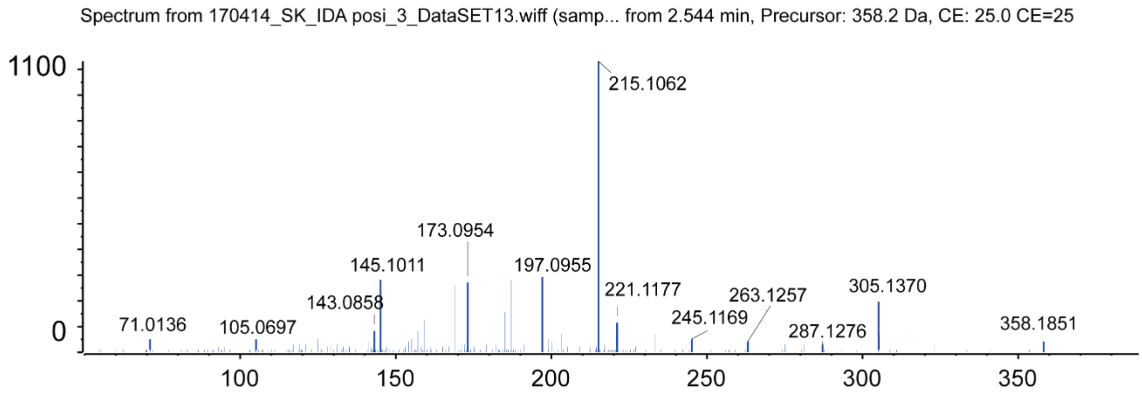

(H) 15-acetyl-T-2 tetraol $[C_{17}H_{24}O_7 + NH_4]^+$ *m/z*: 358.186, RT: 2.13 min

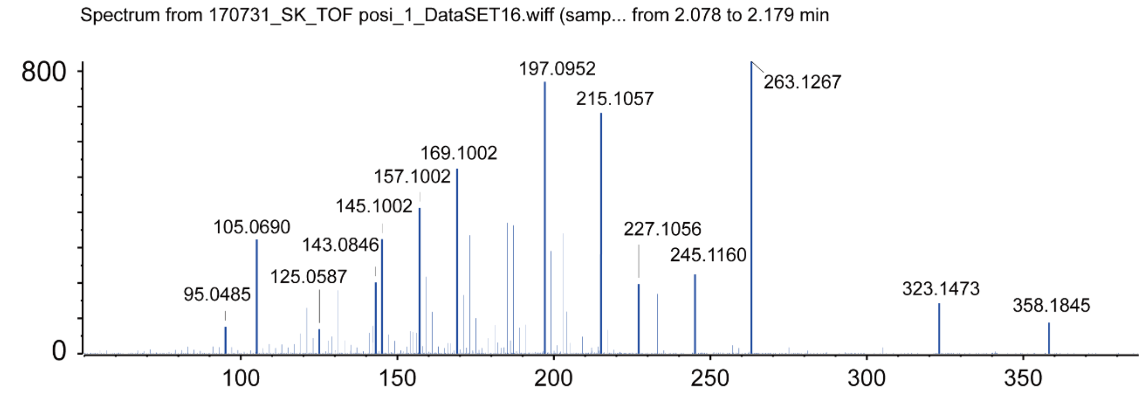

(I-1) 3,15-diacetyl-T-2 tetraol $[C_{19}H_{26}O_8 + H]^+$ *m/z*: 383.170, RT: 2.88 min

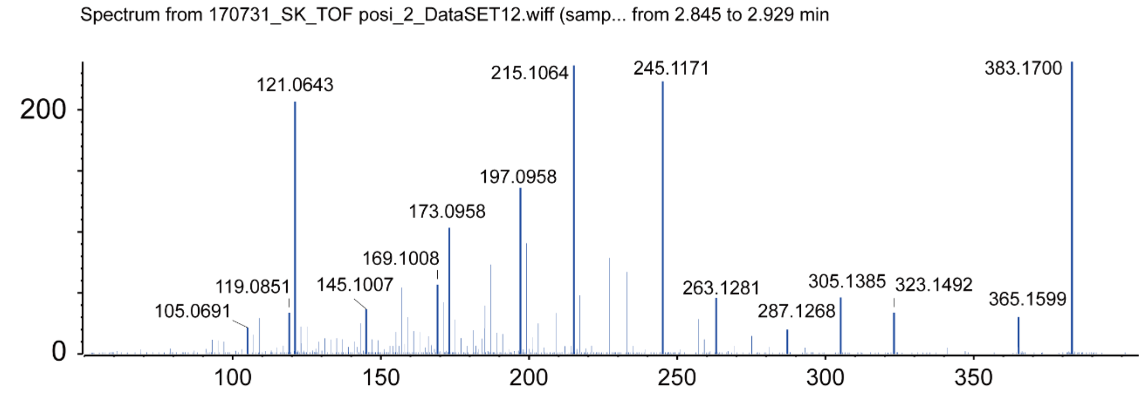

(I-2) 3,15-diacetyl-T-2 tetraol $[C_{19}H_{26}O_8 + NH_4]^+$ *m/z*: 400.197, RT: 2.87 min

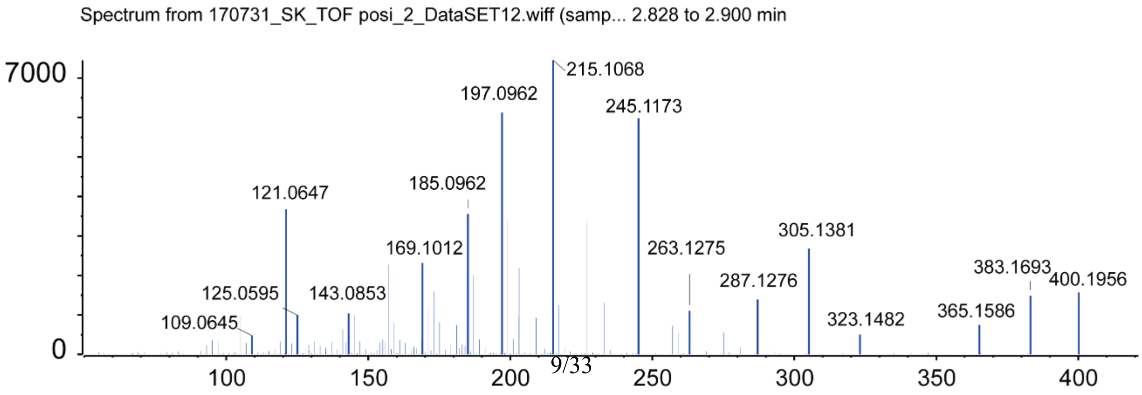

(J-1) 3-acetyl-HT-2 toxin [$C_{24}H_{34}O_9$ + H]$^+$ *m*/*z*: 467.228, RT: 4.52 min

Spectrum from 170414_SK_TOF posi_2_DataSET16.wiff (samp... from 4.476 to 4.602 min

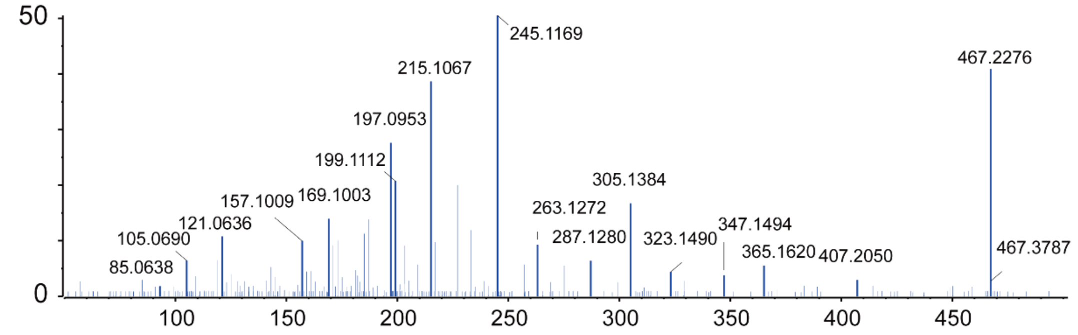

(J-2) 3-acetyl-HT-2 toxin [$C_{24}H_{34}O_9$ + NH_4]$^+$ *m*/*z*: 484.254, RT: 4.52 min

Spectrum from 170414_SK_TOF posi_2_DataSET16.wiff (samp... from 4.471 to 4.573 min

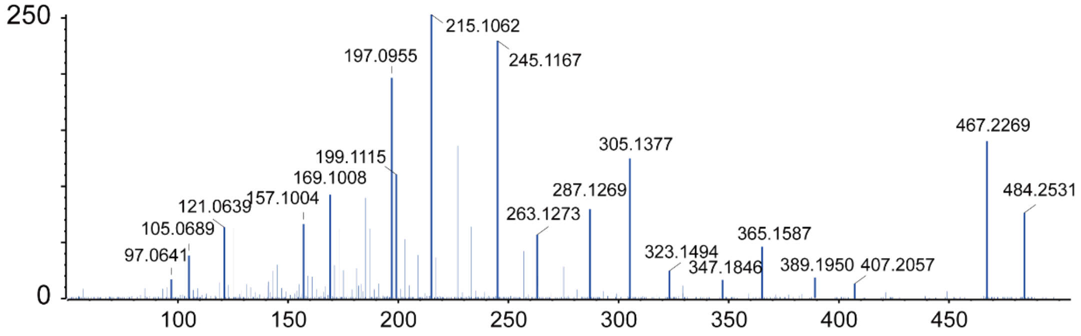

(K-1) scirpenol [$C_{15}H_{22}O_5$ + H]$^+$ *m*/*z*: 283.154, RT: 2.37 min

Spectrum from 170215 _MK_1_TOF posi_DataSET15.wiff (samp... from 2.304 to 2.402 min

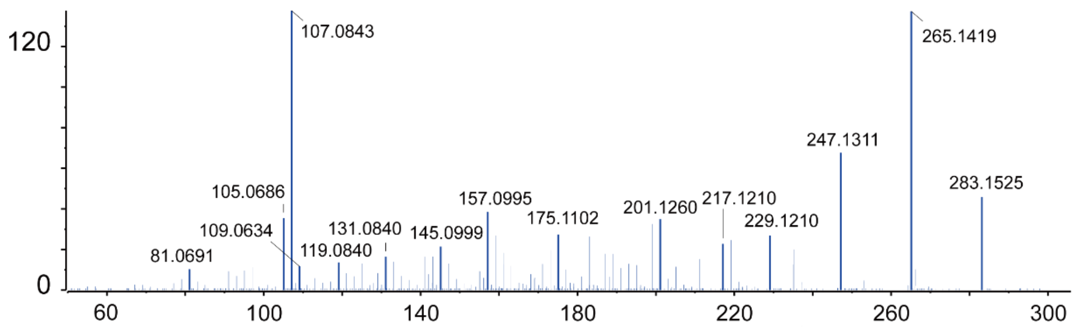

(K-2) scirpenol [$C_{15}H_{22}O_5$ + NH_4]$^+$ *m*/*z*: 300.181, RT: 2.35 min

Spectrum from 170215 _MK_1_TOF posi_DataSET15.wiff (samp... from 2.305 to 2.416 min

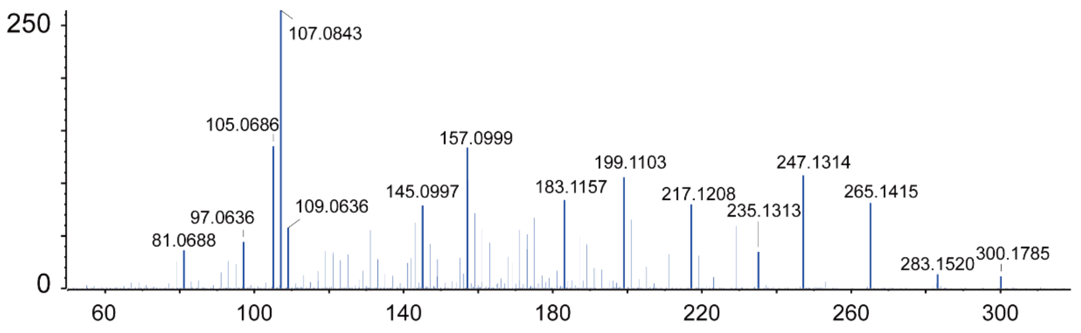

(L) 3-acetoxy-scirpenol $[C_{17}H_{24}O_6 + NH_4]^+$ *m/z*: 342.191, RT: 2.97 min

Spectrum from 170130_MK_2_TOF posi_DataSET15.wiff (samp... from 2.948 to 3.049 min

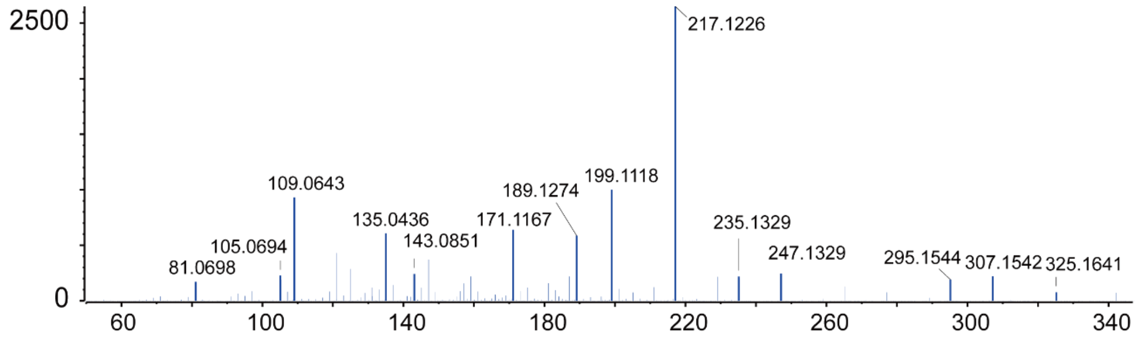

(M) 15-acetoxy-scirpenol $[C_{17}H_{24}O_6 + NH_4]^+$ *m/z*: 342.191, RT: 3.06 min

Spectrum from 170130_MK_2_TOF posi_DataSET16.wiff (samp... from 3.014 to 3.133 min

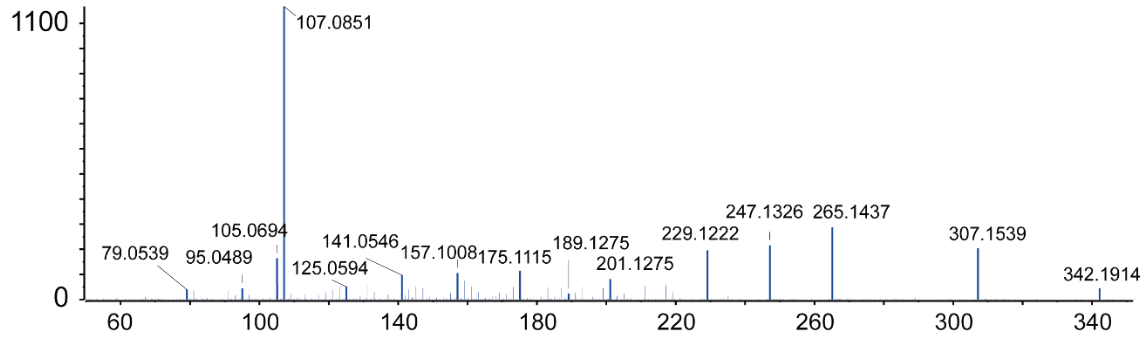

(N-1) 3,15-diacetoxy-scirpenol $[C_{19}H_{26}O_7 + H]^+$ *m/z*: 367.175, RT: 3.63 min

Spectrum from 170130_MK_2_TOF posi_DataSET18.wiff (samp... from 3.564 to 3.635 min

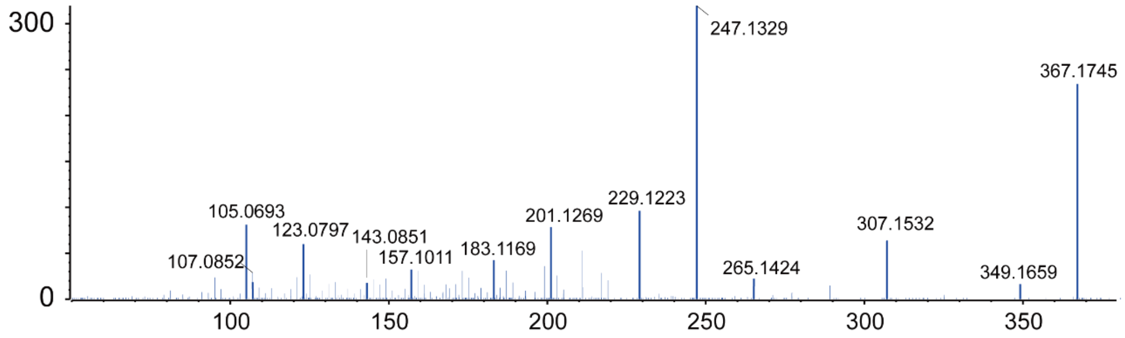

(N-2) 3,15-diacetoxy-scirpenol $[C_{19}H_{26}O_7 + NH_4]^+$ *m/z*: 384.202, RT: 3.60 min

Spectrum from 170130_MK_2_TOF posi_DataSET18.wiff (samp... from 3.547 to 3.732 min

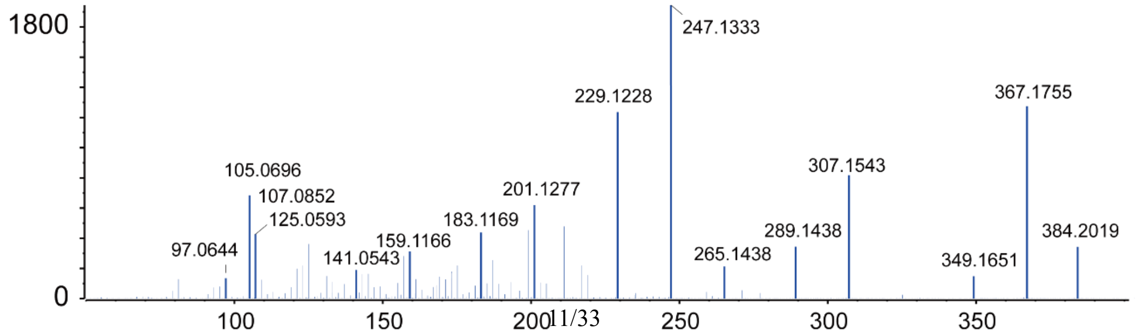

(O-1) 4,15-diacetoxy-scirpenol $[C_{19}H_{26}O_7 + H]^+$ *m/z*: 367.175, RT: 3.70 min

Spectrum from 160913_MK_1_IDAposi_DataSET16.wiff (samp... from 3.698 min, Precursor: 367.2 Da, CE: 25.0 CE=25

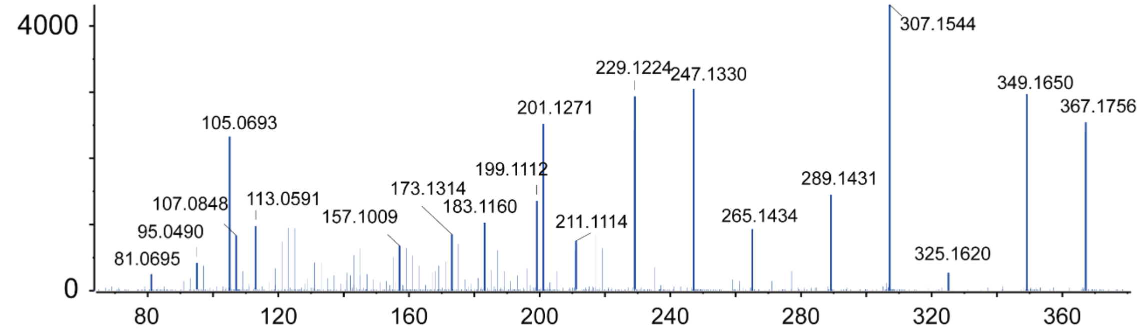

(O-2) 4,15-diacetoxy-scirpenol $[C_{19}H_{26}O_7 + NH_4]^+$ *m/z*: 384.202, RT: 3.70 min

Spectrum from 160913_MK_1_IDAposi_DataSET16.wiff (samp... from 3.771 min, Precursor: 384.2 Da, CE: 25.0 CE=25

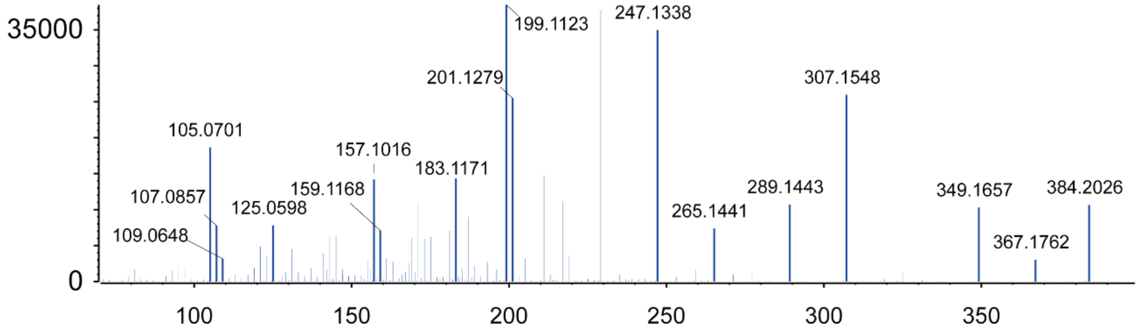

(P) neosolaniol $[C_{19}H_{26}O_8 + NH_4]^+$ *m/z*: 400.197, RT: 2.77 min

Spectrum from 160913_MK_1_IDAposi_DataSET112.wiff (samp...from 2.730 min, Precursor: 400.2 Da, CE: 25.0 CE=25

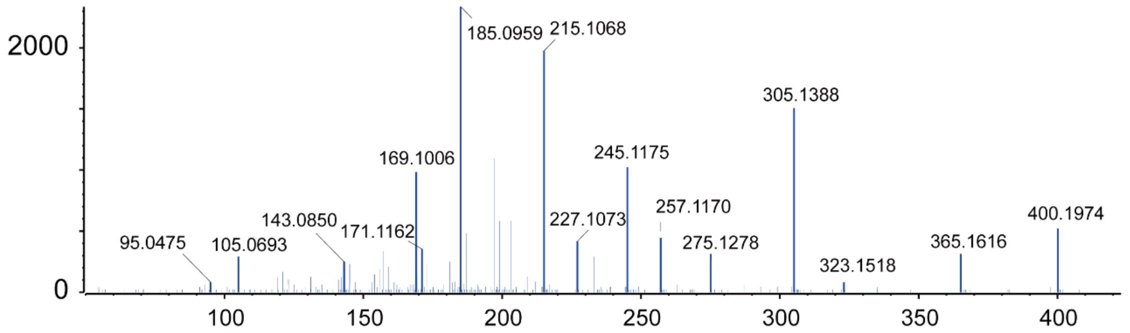

(Q) 3-acetyl-neosolaniol $[C_{21}H_{28}O_8 + NH_4]^+$ *m/z*: 442.207, RT: 3.57 min

Spectrum from 161122_SK_IDA posi_1_DataSET119.wiff (samp... from 3.449 min, Precursor: 442.2 Da, CE: 25.0 CE=25

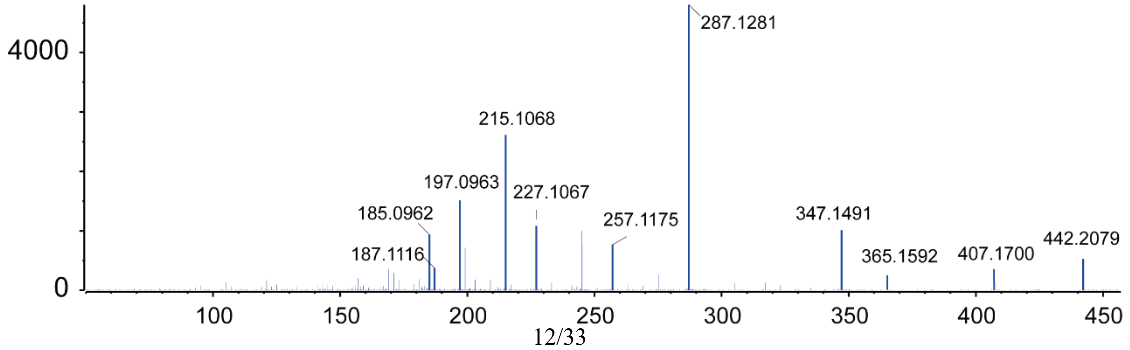

Supplementary Figure S3. LC-MS/MS chromatograms of 17 type A trichothecenes. The purified trichothecenes were subjected to LC-MS analysis, with the MS/MS spectra acquired in positive ion mode using information-dependent acquisition (IDA) and/or time-of-flight (TOF)-MS modes.

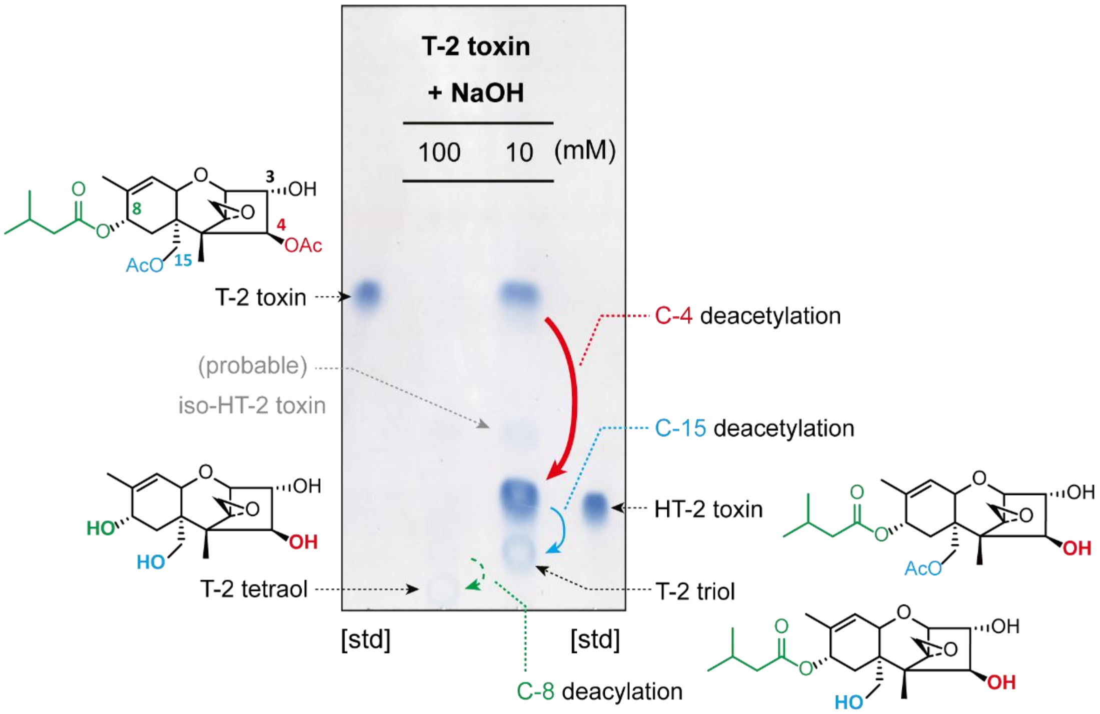

Supplementary Figure S4. Stability of T-2 toxin under alkaline conditions. T-2 toxin was dissolved in 10 mM and 100 mM NaOH, incubated for 10 min at 37 ºC, and then neutralized with HCl. The reaction mixture was diluted with 0.75M Tris-HCl at pH6.8 and analyzed by TLC. Among the three acyl groups attached to the trichothecene skeleton, the 4-*O*-acetyl group exhibited the highest lability.

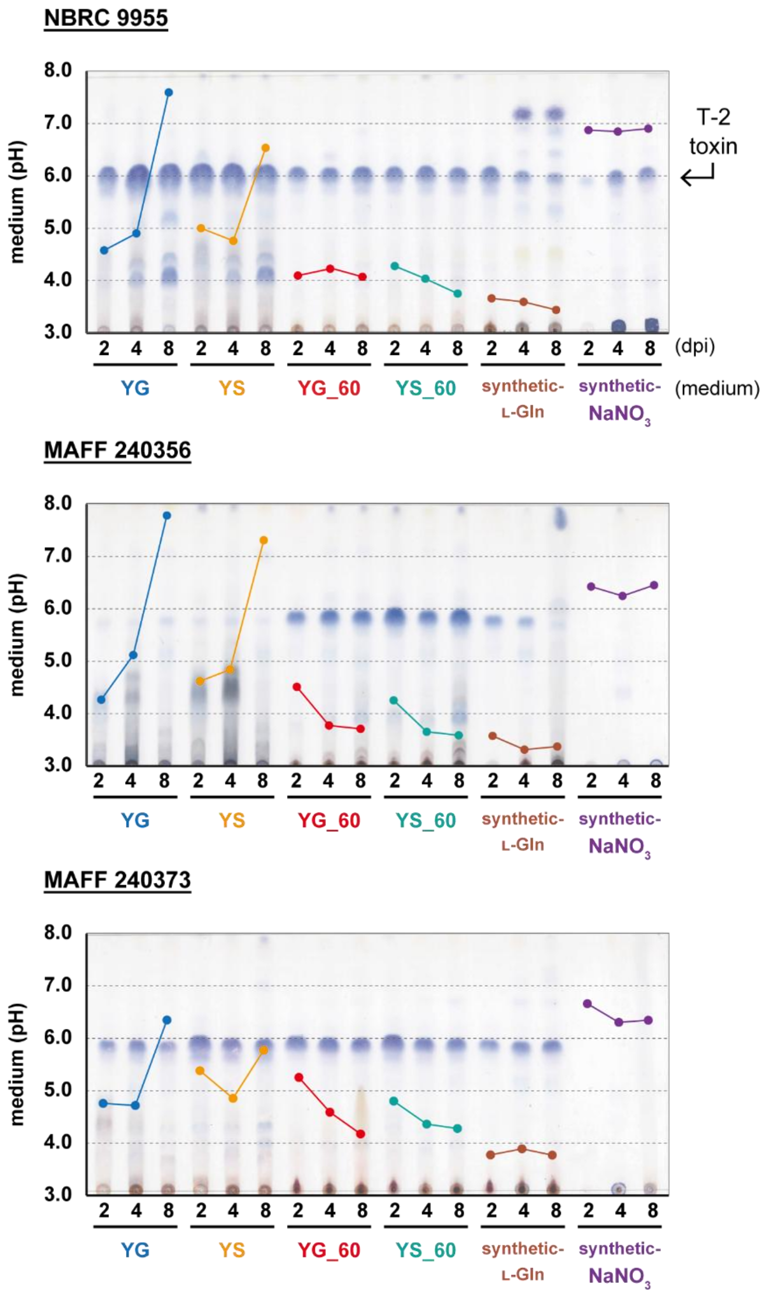

Supplementary Figure S5. Profiles of trichothecene production and culture pH for three *F. sporotrichioides* strains cultivated in various media, including YG, YS, YG_60, YS_60, synthetic glutamine (L-Gln), and synthetic nitrate ($NaNO_3$) media. A one-milliliter sample of the culture extract was analyzed using TLC with a solvent mixture of ethyl acetate/toluene (3:1), monitored up to 8 days post inoculation (dpi).

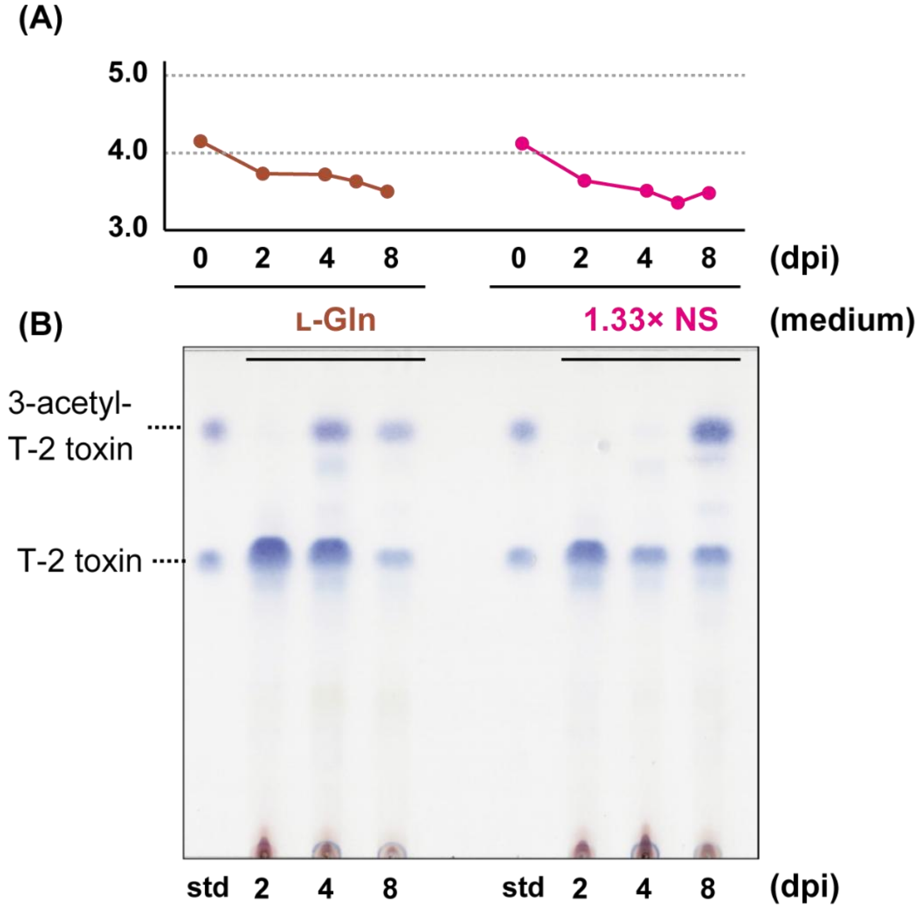

Supplementary Figure S6. Accumulation of 3-acetylT-2 toxin in the NRBC 9955 strain culture using micronutrient-poor synthetic media containing amino acids as the nitrogen source. (A) Temporal changes in pH within the synthetic L-Gln and 1.33 × NS culture media. The 1 × NS medium comprises amino acid mixtures at a total concentration of 3.75 mM [5]. (B) TLC analysis of trichothecenes. A developing solvent, composed of ethyl acetate and toluene in a 3:1 ratio, was employed for separation of the ethyl acetate extracts of the fungal cultures.

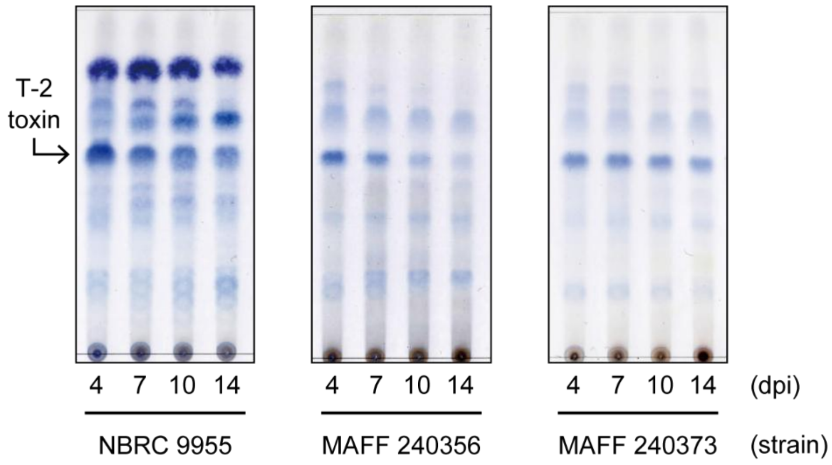

Supplementary Figure S7. TLC analysis of trichothecenes produced by *F. sporotrichioides* strains cultivated on brown rice flour (BRF) solid medium. The ethyl acetate extract, obtained from 0.25 g of the solid culture, was subjected to TLC employing a solvent mixture of ethyl acetate and toluene in a 3:1 ratio.

(A)

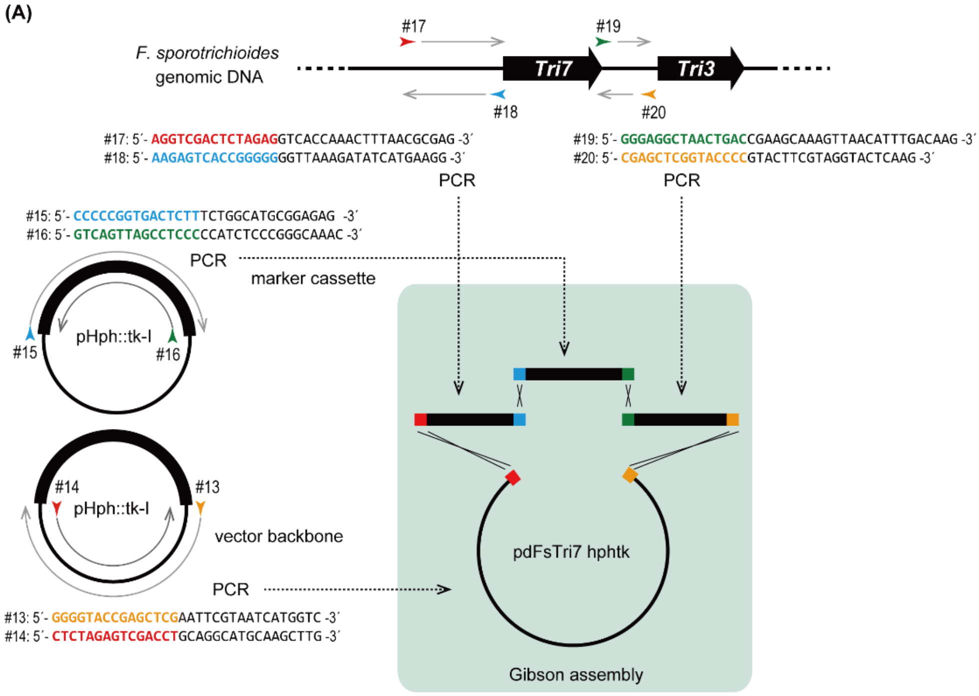
#17
#19
F. sporotrichioides
genomic DNA
Tri7
Tri3
#18
#20
#17: 5′- AGGTCGACTCTAGAGGTCACCAAACTTTAACGCGAG -3′
#18: 5′- AAGAGTCACCGGGGGGGTTAAAGATATCATGAAGG -3′
#19: 5′- GGGAGGCTAACTGACCGAAGCAAAGTTAACATTTGACAAG -3′
#20: 5′- CGAGCTCGGTACCCCGTACTTCGTAGGTACTCAAG -3′
PCR
PCR
#15: 5′- CCCCCGGTGACTCTTTCTGGCATGCGGAGAG -3′
#16: 5′- GTCAGTTAGCCTCCCCCATCTCCCGGGCAAAC -3′
PCR
marker cassette
#15
pHph::tk-I
#16
#14
#13
pHph::tk-I
vector backbone
PCR
pdFsTri7 hphtk
#13: 5′- GGGGTACCGAGCTCGAATTCGTAATCATGGTC -3′
#14: 5′- CTCTAGAGTCGACCTGCAGGCATGCAAGCTTG -3′
Gibson assembly

(B)

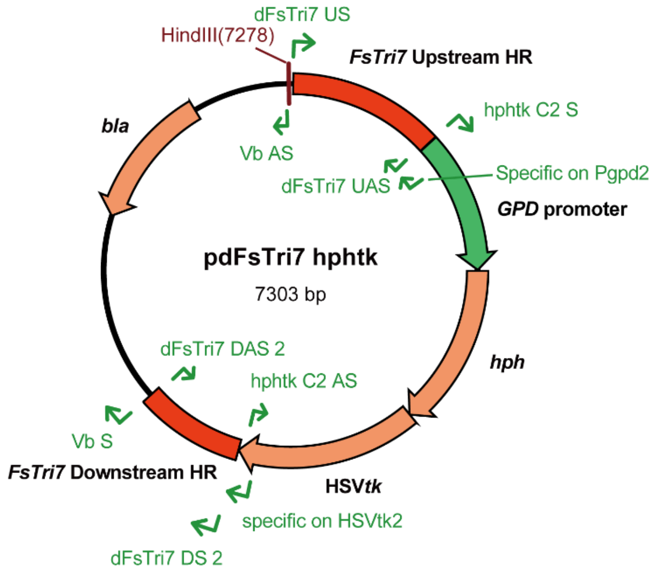
dFsTri7 US
HindIII(7278)
FsTri7 Upstream HR
bla
hphtk C2 S
Vb AS
Specific on Pgpd2
dFsTri7 UAS
GPD promoter
pdFsTri7 hphtk
7303 bp
hph
dFsTri7 DAS 2
hphtk C2 AS
Vb S
FsTri7 Downstream HR
HSVtk
specific on HSVtk2
dFsTri7 DS 2

(C)

FsTri7_Upstream_HR
dFsTri7_US
1 AGAGGTCACC AAACTTTAAC GCGAGCTAGA ACTGATTCAA ATGAGGAAGG GCTGGAAAGT TTCAGATTGC AGACTTGAAA AATACTGTAG TATACTACGT
Vb_AS

FsTri7_Upstream_HR
101 ACTAAAGGGG ACTCAATCTT ACCCCCTTAA CCTTCATTCT TTAGGTAGAG GCTGCAACTG ACTGGCAAAT AACTAGTTGG CTTATAAATA AAAGATACAT

FsTri7_Upstream_HR
201 CTTTCGCTCA GTATAAGAAT TATTGGTAAT TAAATATAGA TTTAATTTAC TGATGAATAG GTCTACATAT CGTTGAGCGC ACTTCGTGTA AAGATGTACA

FsTri7_Upstream_HR
301 ATGAATTTAT TAATAATTCA CTATGCAGTT ATGAGATCTA TATCTATTTA CTAGTAAACT CCATATATAC TGGAGGCACT TTAGGTAAGT TATAAGTGAA

FsTri7_Upstream_HR
401 TTAGCTTGTG CCTGTAGCCT TATTCTGGGG CAGGGTAAAA AAGGTTATGT GTAGGCATCG GAGTCATGAT GGTTTATTGT GAAGTTAAAC ATAAAAGAAA

FsTri7_Upstream_HR
501 TTGGGGGATG AAGGTAAGCT AACTAGCTAT TTGTTGGTCA GTTGCCCCCC CTACTTAAGG AATGAAGGTC AGGGGGGTGA GACTGAAACC CCCCCTAATA

FsTri7_Upstream_HR
601 GAATGAATCT AACTTTATGC AGTGGATTGA GAACGGAAAT TTCGAATTGA TTTCTTGGCC CTAATATGAG CGCAGTTAGA GTTTTAGGTA TAATTGGAAT

FsTri7_Upstream_HR
701 AGTCGCTTTA TTGATACCCT TGTCGGGCCC TGCTGGCCCT GTTATCAGGC CTTCCATTCA CACACTCTAT CTAGTCTACA TACTATCACT GGTTCGTTTC

FsTri7_Upstream_HR
801 GAGATTTAAC TTTGAACTAG GCCCACCCGT GAGGCCTGAG AAAAGTAAGG CTTACTCTGT ATGGTTTTGT CCTTTCTTTC TCTCGCCTGG ACGTTCAGTC

FsTri7_Upstream_HR GPD promoter
hphtk_C2_S
901 TTTGTATATC AAGTGGTTCA TTTTCATGGC CTTCATGATA TCTTTAACCC CCCCGGTGAC TCTTTCTGGC ATGCGGAGAG ACGGACGGAC GCAGAGAGAA
dFsTri7_UAS

GPD promoter
1001 GGGCTGAGTA ATAAGCGCCA CTGCGCCAGA CAGCTCTGGC GGCTCTGAGG TGCAGTGGAT GATTATTAAT CCGGGACCGG CCGCCCCTCC GCCCCGAAGT

GPD promoter
1101 GGAAAGGCTG GTGTGCCCCT CGTTGACCAA GAATCTATTG CATCATCGGA GAATATGGAG CTTCATCGAA TCACCGGCAG TAAGCGAAGG AGAATGTGAA
Specific_on_Pgpd2

GPD promoter
1201 GCCAGGGGTG TATAGCCGTC GGCGAAATAG CATGCCATTA ACCTAGGTAC AGAAGTCCAA TTGCTTCCGA TCTGGTAAAA GATTCACGAG ATAGTACCTT

GPD promoter
1301 CTCCGAAGTA GGTAGAGCGA GTACCCGGCG CGTAAGCTCC CTAATTGGCC CATCCGGCAT CTGTAGGGCG TCCAAATATC GTGCCTCTCC TGCTTTGCCC

GPD promoter
1401 GGTGTATGAA ACCGGAAAGG CCGCTCAGGA GCTGGCCAGC GGCGCAGACC GGGAACACAA GCTGGCAGTC GACCCATCCG GTGCTCTGCA CTCGACCTGC

GPD promoter
1501 TGAGGTCCCT CAGTCCCTGG TAGGCAGCTT TGCCCCGTCT GTCCGCCCGG TGTGTCGGCG GGGTTGACAA GGTCGTTGCG TCAGTCCAAC ATTTGTTGCC

GPD promoter
1601 ATATTTTCCT GCTCTCCCCA CCAGCTGCTC TTTTCTTTTC TCTTTCTTTT CCCATCTTCA GTATATTCAT CTTCCCATCC AAGAACCTTT ATTTCCCCTA

GPD promoter
1701 AGTAAGTACT TTGCTACATC CATACTCCAT CCTTCCCATC CCTTATTCCT TTGAACCTTT CAGTTCGAGC TTTCCCACTT CATCGCAGCT TGACTAACAG

GPD promoter hph
1801 CTACCCCGCT TGAGATCGAT ATGAAAAAGC CTGAACTCAC CGCGACGTCT GTCGAGAAGT TTCTGATCGA AAAGTTCGAC AGCGTCTCCG ACCTGATGCA

hph
1901 GCTCTCGGAG GGCGAAGAAT CTCGTGCTTT CAGCTTCGAT GTAGGAGGGC GTGGATATGT CCTGCGGGTA AATAGCTGCG CCGATGGTTT CTACAAAGAT

hph
2001 CGTTATGTTT ATCGGCACTT TGCATCGGCC GCGCTCCCGA TTCCGGAAGT GCTTGACATT GGGGAGTTCA GCGAGAGCCT GACCTATTGC ATCTCCCGCC

hph
2101 GTGCACAGGG TGTCACGTTG CAAGACCTGC CTGAAACCGA ACTGCCCGCT GTTCTCCAGC CGGTCGCGGA GGCCATGGAT GCGATCGCTG CGGCCGATCT

hph
2201 TAGCCAGACG AGCGGGTTCG GCCCATTCGG ACCGCAAGGA ATCGGTCAAT ACACTACATG GCGTGATTTC ATATGCGCGA TTGCTGATCC CCATGTGTAT

hph
2301 CACTGGCAAA CTGTGATGGA CGACACCGTC AGTGCGTCCG TCGCGCAGGC TCTCGATGAG CTGATGCTTT GGGCCGAGGA CTGCCCCGAA GTCCGGCACC

hph
2401 TCGTGCATGC GGATTTCGGC TCCAACAATG TCCTGACGGA CAATGGCCGC ATAACAGCGG TCATTGACTG GAGCGAGGCG ATGTTCGGGG ATTCCCAATA

hph
2501 CGAGGTCGCC AACATCCTCT TCTGGAGGCC GTGGTTGGCT TGTATGGAGC AGCAGACGCG CTACTTCGAG CGGAGGCATC CGGAGCTTGC AGGATCGCCG

hph
2601 CGCCTCCGGG CGTATATGCT CCGCATTGGT CTTGACCAAC TCTATCAGAG CTTGGTTGAC GGCAATTTCG ATGATGCAGC TTGGGCGCAG GGTCGATGCG

hph
2701 ACGCAATCGT CCGATCCGGA GCCGGGACTG TCGGGCGTAC ACAAATCGCC CGCAGAAGCG CGGCCGTCTG GACCGATGGC TGTGTAGAAG TACTCGCCGA

hph
HSVtk
2801 TAGTGGAAAC CGACGCCCCA GCACTCGTCC GAGGGCAAAG GAAATGGCTT CGTACCCCGG CCATCAACAC GCGTCTGCGT TCGACCAGGC TGCGCGTTCT

HSVtk
2901 CGCGGCCATA GCAACCGACG TACGGCGTTG CGCCCTCGCC GGCAGCAAGA AGCCACGGAA GTCCGCCCGG AGCAGAAAAT GCCCACGCTA CTGCGGGTTT

HSVtk
3001 ATATAGACGG TCCCCACGGG ATGGGGAAAA CCACCACCAC GCAACTGCTG GTGGCCCTGG GTTCGCGCGA CGATATCGTC TACGTACCCG AGCCGATGAC

HSVtk
3101 TTACTGGCGG GTGCTGGGGG CTTCCGAGAC AATCGCGAAC ATCTACACCA CACAACACCG CCTCGACCAG GGTGAGATAT CGGCCGGGGA CGCGGCGGTG

HSVtk
3201 GTAATGACAA GCGCCCAGAT AACAATGGGC ATGCCTTATG CCGTGACCGA CGCCGTTCTG GCTCCTCATA TCGGGGGGGA GGCTGGGAGC TCACATGCCC

HSVtk
3301 CGCCCCCGGC CCTCACCCTC ATCTTCGACC GCCATCCCAT CGCCGCCCTC CTGTGCTACC CGGCCGCGCG GTACCTTATG GGCAGCATGA CCCCCCAGGC

HSVtk
3401 CGTGCTGGCG TTCGTGGCCC TCATCCCGCC GACCTTGCCC GGCACCAACA TCGTGCTTGG GGCCCTTCCG GAGGACAGAC ACATCGACCG CCTGGCCAAA

HSVtk
3501 CGCCAGCGCC CCGGCGAGCG GCTGGACCTG GCTATGCTGG CTGCGATTCG CCGCGTTTAC GGGCTACTTG CCAATACGGT GCGGTATCTG CAGTGCGGCG

HSVtk
3601 GGTCGTGGCG GGAGGACTGG GGACAGCTTT CGGGGACGGC CGTGCCGCCC CAGGGTGCCG AGCCCCAGAG CAACGCGGGC CCACGACCCC ATATCGGGGA

HSVtk
3701 CACGTTATTT ACCCTGTTTC GGGCCCCCGA GTTGCTGGCC CCCAACGGCG ACCTGTATAA CGTGTTTGCC TGGGCCTTGG ACGTCTTGGC CAAACGCCTC

Specific_on_HSVtk2
HSVtk
3801 CGTTCCATGC ACGTCTTTAT CCTGGATTAC GACCAATCGC CCGCCGGCTG CCGGGACGCC CTGCTGCAAC TTACCTCCGG GATGGTCCAG ACCCACGTCA

FsTri7_Downstream_HR
HSVtk
dFsTri7_DS_2
3901 CCACCCCCGG CTCCATACCG ACGATATGCG ACCTGGCGCG CACGTTTGCC CGGGAGATGG GGGAGGCTAA CTGACCGAAG CAAAGTTAAC ATTTGACAAG
hphtk_C2_AS

FsTri7_Downstream_HR
4001 ATATCTAGAT ACCATACCTA GATACTTATA GTAATGCAGC TGATTGTTCT CTCCATGACA AGAAGGTGCT CGATGCTAGA TGAGATAGGA GCGACGACAT

FsTri7_Downstream_HR
4101 GAATATTCTA GTATGTAAAA AATGCATCCC TGGCTTCAAG TGCATTAAAT TGGATCCTTC AAGCGTTATG CGTTCTTTCA TGACGAGCAC CATGAATTAG

FsTri7_Downstream_HR
4201 TTATCTTCCC TATAGGCCGC CCGATGGTAC TACGACACAC ATGTGACCCT TGACCAATGT ATCGCGGTTG TGATGCCAGG AGCTAACTGC TAAAGAAATA

FsTri7_Downstream_HR
4301 CGATAGGCAT TCTGTCCGTC TAGCCAACTT GAACTCACCG TGGGGGTAGG GAAGTAAGAG GTGCAAGGGT GAATGTTTTG ATACGCTTGC TCCAGACTAA

FsTri7_Downstream_HR
4401 GAATCGAACT CTGAGAGCGG GTTATCGGTT CTATAGAAAG AAGTTAAGGC GTGTCAGTGG AGGCTGTGTA AGGCCCGACA GTGCATTGTA GGCTAAGACT

FsTri7_Downstream_HR
4501 GTCGGGGTCT CAATACCTTG TTAGTTTGCA TTATATGGAA CATTTGAAAG TCAATGTTTT ACGAAATGTG TTAGTTCTGT TTGCAAAAGG GCGCCTGTAT

FsTri7_Downstream_HR
Vb_S
4601 TTATTATCTT GAGTACCTAC GAAGTACGGG GTACCGAGCT CGAATTCGTA ATCATGGTCA TAGCTGTTTC CTGTGTGAAA TTGTTATCCG CTCACAATTC
dFsTri7_DAS_2

4701 CACACAACAT ACGAGCCGGA AGCATAAAGT GTAAAGCCTG GGGTGCCTAA TGAGTGAGCT AACTCACATT AATTGCGTTG CGCTCACTGC CCGCTTTCCA
4801 GTCGGGAAAC CTGTCGTGCC AGCTGCATTA ATGAATCGGC CAACGCGCGG GGAGAGGCGG TTTGCGTATT GGGCGCTCTT CCGCTTCCTC GCTCACTGAC
4901 TCGCTGCGCT CGGTCGTTCG GCTGCGGCGA GCGGTATCAG CTCACTCAAA GGCGGTAATA CGGTTATCCA CAGAATCAGG GGATAACGCA GGAAAGAACA
5001 TGTGAGCAAA AGGCCAGCAA AAGGCCAGGA ACCGTAAAAA GGCCGCGTTG CTGGCGTTTT TCCATAGGCT CCGCCCCCCT GACGAGCATC ACAAAAATCG
5101 ACGCTCAAGT CAGAGGTGGC GAAACCCGAC AGGACTATAA AGATACCAGG CGTTTCCCCC TGGAAGCTCC CTCGTGCGCT CTCCTGTTCC GACCCTGCCG
5201 CTTACCGGAT ACCTGTCCGC CTTTCTCCCT TCGGGAAGCG TGGCGCTTTC TCATAGCTCA CGCTGTAGGT ATCTCAGTTC GGTGTAGGTC GTTCGCTCCA
5301 AGCTGGGCTG TGTGCACGAA CCCCCCGTTC AGCCCGACCG CTGCGCCTTA TCCGGTAACT ATCGTCTTGA GTCCAACCCG GTAAGACACG ACTTATCGCC
5401 ACTGGCAGCA GCCACTGGTA ACAGGATTAG CAGAGCGAGG TATGTAGGCG GTGCTACAGA GTTCTTGAAG TGGTGGCCTA ACTACGGCTA CACTAGAAGG
5501 ACAGTATTTG GTATCTGCGC TCTGCTGAAG CCAGTTACCT TCGGAAAAAG AGTTGGTAGC TCTTGATCCG GCAAACAAAC CACCGCTGGT AGCGGTGGTT
5601 TTTTTGTTTG CAAGCAGCAG ATTACGCGCA GAAAAAAAGG ATCTCAAGAA GATCCTTTGA TCTTTTCTAC GGGGTCTGAC GCTCAGTGGA ACGAAAACTC
5701 ACGTTAAGGG ATTTTGGTCA TGAGATTATC AAAAAGGATC TTCACCTAGA TCCTTTTAAA TTAAAAATGA AGTTTTAAAT CAATCTAAAG TATATATGAG
5801 TAAACTTGGT CTGACAGTTA CCAATGCTTA ATCAGTGAGG CACCTATCTC AGCGATCTGT CTATTTCGTT CATCCATAGT TGCCTGACTC CCCGTCGTGT
bla

5901 AGATAACTAC GATACGGGAG GGCTTACCAT CTGGCCCCAG TGCTGCAATG ATACCGCGAG ACCCACGCTC ACCGGCTCCA GATTTATCAG CAATAAACCA
bla

6001 GCCAGCCGGA AGGGCCGAGC GCAGAAGTGG TCCTGCAACT TTATCCGCCT CCATCCAGTC TATTAATTGT TGCCGGGAAG CTAGAGTAAG TAGTTCGCCA
bla

```
6101  GTTAATAGTT TGCGCAACGT TGTTGCCATT GCTACAGGCA TCGTGGTGTC ACGCTCGTCG TTTGGTATGG CTTCATTCAG CTCCGGTTCC CAACGATCAA
                                                  bla
6201  GGCGAGTTAC ATGATCCCCC ATGTTGTGCA AAAAAGCGGT TAGCTCCTTC GGTCCTCCGA TCGTTGTCAG AAGTAAGTTG GCCGCAGTGT TATCACTCAT
                                                  bla
6301  GGTTATGGCA GCACTGCATA ATTCTCTTAC TGTCATGCCA TCCGTAAGAT GCTTTTCTGT GACTGGTGAG TACTCAACCA AGTCATTCTG AGAATAGTGT
                                                  bla
6401  ATGCGGCGAC CGAGTTGCTC TTGCCCGGCG TCAATACGGG ATAATACCGC GCCACATAGC AGAACTTTAA AAGTGCTCAT CATTGGAAAA CGTTCTTCGG
                                                  bla
6501  GGCGAAAACT CTCAAGGATC TTACCGCTGT TGAGATCCAG TTCGATGTAA CCCACTCGTG CACCCAACTG ATCTTCAGCA TCTTTTACTT TCACCAGCGT
                                                  bla
6601  TTCTGGGTGA GCAAAAACAG GAAGGCAAAA TGCCGCAAAA AAGGGAATAA GGGCGACACG GAAATGTTGA ATACTCATAC TCTTCCTTTT TCAATATTAT
                                       bla
6701  TGAAGCATTT ATCAGGGTTA TTGTCTCATG AGCGGATACA TATTTGAATG TATTTAGAAA AATAAACAAA TAGGGGTTCC GCGCACATTT CCCCGAAAAG
6801  TGCCACCTGA CGTCTAAGAA ACCATTATTA TCATGACATT AACCTATAAA AATAGGCGTA TCACGAGGCC CTTTCGTCTC GCGCGTTTCG GTGATGACGG
6901  TGAAAACCTC TGACACATGC AGCTCCCGGA GACGGTCACA GCTTGTCTGT AAGCGGATGC CGGGAGCAGA CAAGCCCGTC AGGGCGCGTC AGCGGGTGTT
7001  GGCGGGTGTC GGGGCTGGCT TAACTATGCG GCATCAGAGC AGATTGTACT GAGAGTGCAC CATATGCGGT GTGAAATACC GCACAGATGC GTAAGGAGAA
7101  AATACCGCAT CAGGCGCCAT TCGCCATTCA GGCTGCGCAA CTGTTGGGAA GGGCGATCGG TGCGGGCCTC TTCGCTATTA CGCCAGCTGG CGAAAGGGGG
                                                                                 HindIII
7201  ATGTGCTGCA AGGCGATTAA GTTGGGTAAC GCCAGGGTTT TCCCAGTCAC GACGTTGTAA AACGACGGCC AGTGCCAAGC TTGCATGCCT GCAGGTCGAC
                                                                                               Vb_AS
7301  TCT
      Vb_AS
```

Supplementary Figure S8. Construction of *Tri7* gene disruption vector, pdFsTri7_hphtk. (**A**) Strategy of pdFsTri7_hphtk vector construction. The DNA fragments in the vector were amplified using PCR primers with 15 bp overhangs necessary for Gibson assembly, as depicted in the figure (see also legend of Supplementary Table S4). The vector backbone and *hph* cassette (fused with HSV*tk*) were amplified from pHph::tk-I using primer combinations #13 × #14 and #15 × #16, respectively [6]. The upstream and downstream regions of *Tri7* were amplified from the genomic DNA of strain NBRC 9955 using primers #17 × #18 and #19 × #20, respectively. The four DNA fragments were assembled using the NEBuilder® HiFi DNA Assembly Master Mix (New England Biolabs Japan Inc., Tokyo, Japan). (**B**) Map of pdFsTri7_hphtk. The primer names are shown in green. A unique *Hin*dIII site, highlighted in red, was used for vector linearization. (**C**) DNA sequence of pdFsTri7_hphtk.

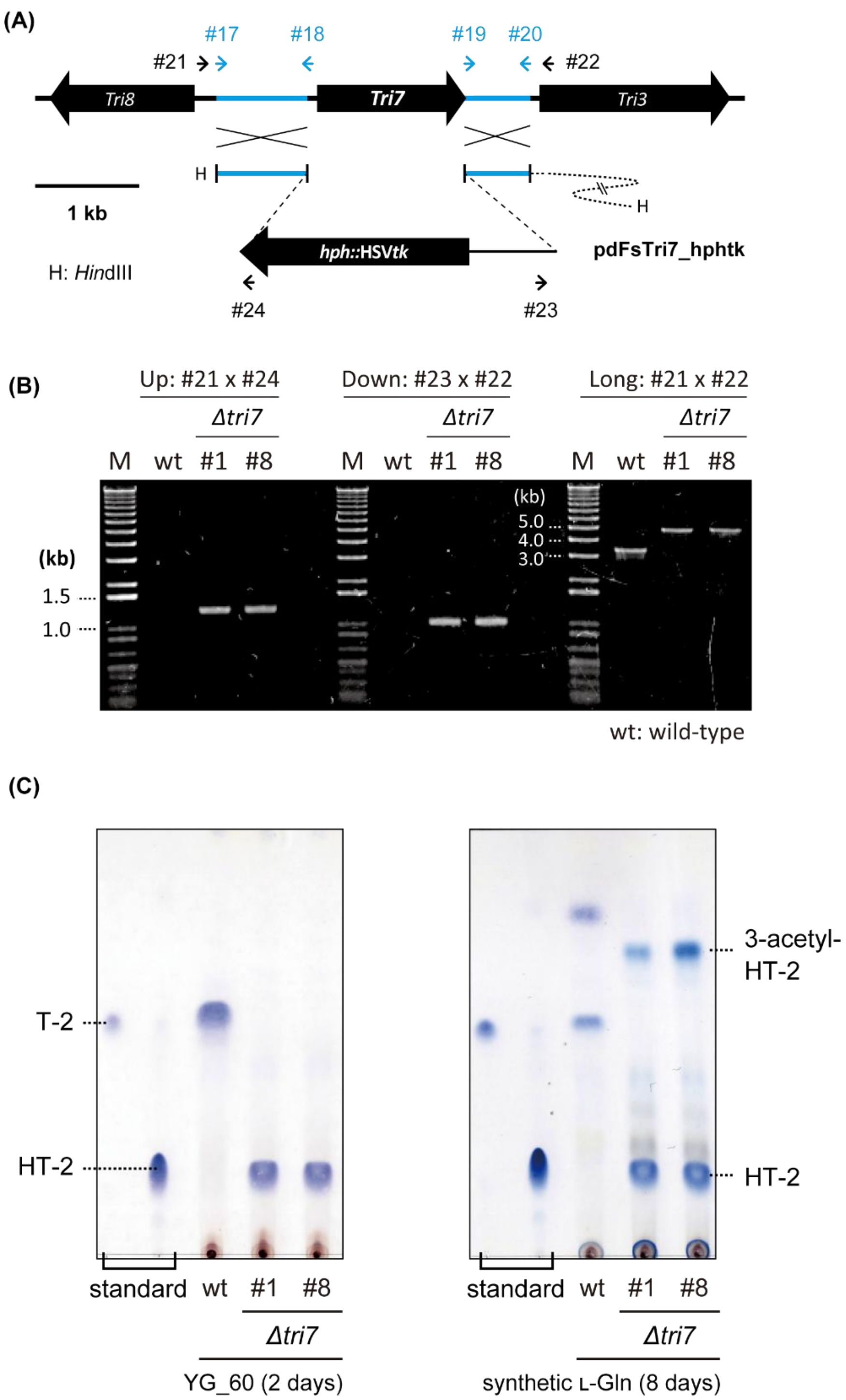

Supplementary Figure S9. Validation and characterization of *Tri7* gene disruption mutants of *F. sporotrichioides* NBRC 9955. (**A**) Scheme for the generation of *Tri7* gene disruption mutants. The transgenic strains (#1 and #8) were produced via transformation with pdFsTri7_hphtk (Supplementary Figure S8) and selection of double cross-over

homologous recombinants through positive selection with hygromycin B. (**B**) PCR validation of transformants. The primer pairs #21 × #24 and #22 × #23 specifically amplify the junction between the integrated vector pdFsTri7_hphtk and the genomic region outside the homologous area (indicated in blue); the primer pair #21 x #22 amplifies the fragment encompassing the deleted region. The expected bands indicative of the *tri7* deletion were observed when primers #21 × #24 (1.2 kb), #22 × #23 (1.0 kb), and #21 × #22 (4.8 kb) were employed. (**C**) Trichothecene profiles of the transformants. The fungal strains were cultured in YG_60 and synthetic L-glutamine (L-Gln) media for 2 and 8 days, respectively, prior to metabolite analyses via TLC using ethyl acetate:toluene (3:1) as the developing solvent. When the Δ*tri7* strain was cultivated in the YG_60 medium, HT-2 toxin was produced. However, when the medium was substituted with the L-Gln medium, an additional formation of 3-acetylHT-2 toxin was observed.

Supplementary Table S1. *Fusarium* strains and accession numbers of their *RPB1*, *RPB2,* and *EF1α* sequences used in molecular phylogenetic analysis.

**Species**	**Strain name** [a]	**DDBJ/GenBank/EMBL accession no.**			**References**
		**RPB1**	**RPB2**	**EF1α**	
Fusarium sporotrichioides	NBRC 9955 [b]	LC909054	LC909055	LC269015	This study
F. armeniacum	FRC R6472	MW233328	MW233500	MW233156	[7]
	FRC R7479	MW233340	MW233512	MW233168	[7]
	FRC R8519	MW233363	MW233535	MW233191	[7]
	FRC R8609	MW233364	MW233536	MW233192	[7]
	NRRL 13343	MW233230	MW233402	MW233059	[7]
F. goolgardi	KOD 1087	MW233390	MW233562	MW233218	[7]
	KOD 1090	MW233391	MW233563	MW233219	[7]
F. langsethiae	NRRL 34176	MW233283	MW233455	MW233111	[7]
	NRRL 36236	MW233286	MW233458	MW233114	[7]
	NRRL 54940	MW233310	MW233482	MW233138	[7]
F. nelsonii	NRRL 13338	MW233397	MW233569	MW233225	[7]
F. nodosum	NRRL 13431	MW233236	MW233408	MW233065	[7]
	NRRL 36351	MW233289	MW233461	MW233117	[7]
F. palustre	NRRL 43289	MW233296	MW233468	MW233124	[7]
	NRRL 54056	MW233303	MW233475	MW233131	[7]
	NRRL 54058	MW233304	MW233476	MW233132	[7]
F. sibiricum	NRRL 53429	MW233301	MW233473	MW233129	[7, 8]

	NRRL 53430	MW233302	MW233474	MW233130	[7, 8]
F. sporotrichioides	NRRL 13440	MW233237	MW233409	MW233066	[7, 8]
	NRRL 25474	MW233252	MW233424	MW233081	[7, 8]
	NRRL 26923	MW233260	MW233432	MW233089	[7, 8]
	NRRL 29131	MW233268	MW233440	MW233096	[7-9]
	NRRL 3299	MW233226	MW233398	MW233055	[7, 8]
	NRRL 36295	MW233288	MW233460	MW233116	[7]
	NRRL 52731	MW233300	MW233472	MW233128	[7, 8]
F. chaquense	FRC R3766	MW233317	MW233489	MW233145	[4, 7]
	FRC R8102	MW233353	MW233525	MW233181	[4, 7]
Fusarium sp. FSAMSC2	FRC R5100	MW233323	MW233495	MW233151	[4, 7]
	NRRL 54062	MW233305	MW233477	MW233133	[4, 7]
F. parabolicum	FRC R8090	MW233352	MW233524	MW233180	[4, 7]
	FRC R9068	MW233381	MW233553	MW233209	[4, 7]
F. leptum	NRRL 29896	MW233272	MW233444	MW233100	[4, 7]
	NRRL 29897	MW233273	MW233445	MW233101	[4, 7]

[a] NBRC, National Institute of Technology and Evaluation Biological Resource Center; FRC, Fusarium Research Center, The Pennsylvania State University, University Park, Pennsylvania; KOD, available from Kerry O'Donnell.; NRRL, Northern Regional Research Laboratory

[b] Incorrectly identified and listed as *F. solani* in NBRC culture collection (as of December 2025).

Table S2. Conidiogenesis related gene orthologues.

Locus_tag (FSPOR_) [a]	Description [a]	Length (aa)	Identity [b] (%)	Substitutions [b]	Ortholog in *Fg* (FGRAMPH1_)	FGSG_id	Gene name [c]	References
9644	Homeobox transcription	527	99.6	V449L, A497T	01G23953	FGSG_07097	Htf1	[10, 11]
11915, 7128	Developmental regulatory protein wetA	608	98.6	Differences in His/Pro-rich region	01G07441	FGSG_17727	WetA	[12]
5317	Regulatory abaa	860	99.9	D444E	01G02219	FGSG_11850	AbaA	[13]
1067	Myblike dna-binding	287	99.6	A234V	01G04623	FGSG_01915	FlbD	[14]
9888	Hypothetical protein (related to transcription factor medusa)	728	100	-	01G05927	FGSG_02471	Med1	[15]
7862	Hypothetical protein (related to ascospore maturation 1 asm-1)	550	100	-	01G07355	FGSG_10129	StuA	[16]
8798	Hypothetical protein (related to transcriptional regulator)	420	100	-	01G14573	FGSG_04134	Con7	[17]
2800	Ap-1 complex subunit sigma-1	155	100	-	01G07127	FGSG_10034	FgAP1σ	[18]
6395	Bb56-delta regulatory subunit of phosphatase 2a	655	100	-	01G19019	FGSG_05894	Rts1	[19]
10929	Tyrosine-protein phosphatase yvh1	458	98.5	Q5R, E18A,K26E, V35D, T54A, T241P, L426I	01G08303	FGSG_10516	Yvh1	
1913	Trehalose 6-phosphate synthase	886	100	-	01G25999	FGSG_07926	Tps2	
2917	Plasma membrane phosphatase required for sodium stress response	486	99.4	A119T, P232L, L297S	01G03731	FGSG_01527	Psr2	
2380	Phosphatidylglycerophosphatase mitochondrial	520	96.5	W113X (opal mutation), and 16 aa substitutions	01G19531	FGSG_12867	Gep4	

[a] The orthologue was screened by using an annotated GenBank assembly, GCA_003012315.1 (*F. sporotrichioides* NRRL 3299).

[b] The amino acid sequence of the protein in NBRC 9955 was predicted using the whole genome shotgun sequence (deposited as DRR787368) by referencing to the orthologous gene of *F. sporotrichioides* NRRL 3299, employing the GENETYX-NGS software (version 4.1.1).

[c] The gene name was derived from an annotated GenBank assembly, GCA_900044135.1 (*F. graminearum* strain PH-1) in the Ensembl Fungi database (https://fungi.ensembl.org/index.html) or individual references.

The utility of the short sequence read archive (SRA) for strain NBRC 9955 was evaluated by screening for mutations responsible for the observed shorter and wider conidia in CMC liquid medium (Supplementary Figure S2B). Candidate genes previously implicated in conidiogenesis in the model fungus *Fusarium graminearum* were selected. Using the known orthologous sequences of *F. sporotrichioides* NRRL 3299 as a reference, the corresponding gene sequences of strain NBRC 9955 were assembled from the short paired-end reads. Among the 13 genes analyzed, a nonsense mutation (Trp-113 to opal mutation) was identified in the *Gep4* phosphatase gene (FGSG_12867/FSPOR_2380), which, when deleted, results in the production of shorter conidia with fewer septa [19]. This provides an initial clue for analyzing the unique features of strain NBRC 9955, thereby exemplifying the utility of the SRA. Complementation experiments with the wild-type gene (a functional orthologue of other strains) may provide further insights into the genetic basis underlying the unique morphology observed in *F. sporotrichioides* NBRC 9955.

Supplementary Table S3. Composition of synthetic liquid and BRF solid media.

Synthetic liquid medium for submerged culture

Element	Concentration (/liter)
Sucrose	30 g
KH_2PO_4	1.0 g
KCl	0.5 g
$MgSO_4 \cdot 7H_2O$	0.5 g
Trace elements [1, 2]	0.2 mL
$FeSO_4 \cdot 7H_2O$	0.01 g
Nitrogen source [1, 2, 3]	5 mM

[1] added after autoclave.
[2] filter-sterilized.
[3] L-glutamine or sodium nitrate.

5000 × Trace elements [5]

Element	Concentration (/100 mL)
Citric acid	5.0 g
$MnSO_4$	0.05 g
$ZnSO_4 \cdot 6H_2O$	5.0 g
H_3BO_3	0.05 g
$Na_2MoO_4 \cdot 2H_2O$	0.05 g
$CuSO_4 \cdot 5H_2O$	0.25 g

BRF solid medium [20]

Element	(/bottle)
Brown rice	5.0 g
Distilled water	4.5 mL

BRF solid medium was prepared by mixing 5 g of crushed brown rice with water (4.5 mL), allowing it to stand at room temperature for 3 h, and then autoclaved at 121°C for 20 min.

Supplementary Table S4. Primers used in this study.

No.	Primer	Sequence (5'-3') [a]	Description	References
#01	Fa	CAYAARGARTCYATGATGGGWC	Amplification and sequencing of partial sequence of *RPB1*	[21]
#02	G2R	GTCATYTGDGTDGCDGGYTCDCC	Amplification and sequencing of partial sequence of *RPB1*	[21]
#03	R8	CAATGAGACCTTCTCGACCAGC	Sequencing of partial sequence of *RPB1*	[21]
#04	F5	ATGGGTATYGTCCAGGAYTC	Sequencing of partial sequence of *RPB1*	[21]
#05	F7	CRACACAGAAGAGTTTGAAGG	Sequencing of partial sequence of *RPB1*	[21]
#06	F8	TTCTTCCACGCCATGGCTGGTCG	Sequencing of partial sequence of *RPB1*	[21]
#07	5f2	GGGGWGAYCAGAAGAAGGC	Amplification and sequencing of partial sequence of *RPB2*	[21]
#08	7cr	CCCATRGCTTGYTTRCCCAT	Amplification and sequencing of partial sequence of *RPB2*	[21]
#09	7cf	ATGGGYAARCAAGCYATGGG	Amplification and sequencing of partial sequence of *RPB2*	[21]
#10	11ar	GCRTGGATCTTRTCRTCSACC	Amplification and sequencing of partial sequence of *RPB2*	[21]
#11	EF1	ATGGGTAAGGARGACAAGAC	Amplification and sequencing of partial sequence of *EF1α*	[22]
#12	EF2	GGARGTACCAGTSATCATGTT	Amplification and sequencing of partial sequence of *EF1α*	[22]
#13	Vb_S	GGGGTACCGAGCTCGAATTCGTAATCATGGTC	Construction of pdFsTri7_hphtk	[23]
#14	Vb_AS	CTCTAGAGTCGACCTGCAGGCATGCAAGCTTG	Construction of pdFsTri7_hphtk	[23]
#15	hphtkC2_S	CCCCCGGTGACTCTTTCTGGCATGCGGAGAG	Construction of pdFsTri7_hphtk	[23]
#16	hphtkC2_AS	GTCAGTTAGCCTCCCCCATCTCCCGGGCAAAC	Construction of pdFsTri7_hphtk	[23]
#17	dFsTri7_US	AGGTCGACTCTAGAGGTCACCAAACTTTAACGCGAG	Construction of pdFsTri7_hphtk	This study
#18	dFsTri7_UAS	AAGAGTCACCGGGGGGGTTAAAGATATCATGAAGG	Construction of pdFsTri7_hphtk	This study
#19	dFsTri7_DS_2	GGGAGGCTAACTGACCGAAGCAAAGTTAACATTTGACAAG	Construction of pdFsTri7_hphtk	This study
#20	dFsTri7_DAS_2	CGAGCTCGGTACCCCGTACTTCGTAGGTACTCAAG	Construction of pdFsTri7_hphtk	This study

#21	dFsTri7_LUS_2	GTGAGAGATGCGACAGACCATCATGTTCAG	Screening of *Tri7* gene disruption mutants	This study
#22	dFsTri7_LDAS	TTCTCTTCTAGTCCAACAATCGCTTCAGCG	Screening of *Tri7* gene disruption mutants	This study
#23	Specific_on_Pgpd2	TGATGCAATAGATTCTTGGTCAACGAGGGGC	Screening of *Tri7* gene disruption mutants	[24]
#24	Specific_on_HSVtk2	GCACGTCTTTATCCTGGATTACGACCAATCG	Screening of *Tri7* gene disruption mutants	[24]
#25	FsTri5_RT-Fw	TCCTTAACACTAGCGTGCGCC	Preparation of *Tri5* probe for Northern blot analysis	This study
#26	FsTri5_RT-Rev	GACCCAAGCCGTTCATACGTC	Preparation of *Tri5* probe for Northern blot analysis	This study
#27	FsTri4_RT-Fw	CGCCAACATGTTCAGTCTTGC	Preparation of *Tri4* probe for Northern blot analysis	This study
#28	FsTri4_RT-Rev	GAATGCTCGATGAGGGTACCG	Preparation of *Tri4* probe for Northern blot analysis	This study
#29	FsTri101_RT-F	CATAGAGCTCGACCTTATCGG	Preparation of *Tri101* probe for Northern blot analysis	This study
#30	FsTri101_RT-R	TAGGTCATGCATGTACGTCGC	Preparation of *Tri101* probe for Northern blot analysis	This study
#31	FsTri8_RT-F	GCCATTTGGGAACGCCGTGAT	Preparation of *Tri8* probe for Northern blot analysis	This study
#32	FsTri8_RT-R	ACTCTGCCGATGTCGATGCCA	Preparation of *Tri8* probe for Northern blot analysis	This study

[a] Underlined sequence indicates creation of homologous region (#17 - #20) for construction of pdFsTri7_hphtk

References

1. Domsch, K.H.; Gams, W.; Anderson, T.H., *Compendium of soil fungi*. Academic Press (London) Ltd.: London, UK, 1980; Vol. 1, pp. 338–339.
2. Leslie, J.F.; Summerell, B.A., Media — Recipes and Preparation. In *The Fusarium Laboratory Manual*, 1st ed.; Blackwell Publishing: Ames, IO, USA, 2006; Vol. 1, pp. 3–14.
3. Torp, M.; Nirenberg, H.I., *Fusarium langsethiae* sp. nov. on cereals in Europe. *Int. J. Food Microbiol.* **2004**, *95*, 247–256.
4. Sandoval-Denis, M.; Costa, M.M.; Broders, K.; Becker, Y.; Maier, W.; Yurkov, A.; Kermode, A.; Buddie, A.G.; Ryan, M.J.; Schumacher, R.K.; Groenewald, J.Z.; Crous, P.W., An integrative re-evaluation of the *Fusarium sambucinum* species complex. *Stud. Mycol.* **2025**, *110*, 1–110.
5. Nakao, A.; Nakajima, Y.; Akasaka, M.; Kitou, Y.; Maeda, K.; Kanamaru, K.; Kobayashi, T.; Kimura, M., Synthetic liquid media for the study of trichothecene biosynthesis regulation in *Fusarium graminearum*. *JSM Mycotoxins* **2020**, *70*, 57–59.
6. Maeda, K.; Ohsato, S., Molecular genetic characterization of *Fusarium graminearum* genes identified as encoding a precocene II-binding protein. *JSM Mycotoxins* **2017**, *67*, 1–3.
7. Laraba, I.; McCormick, S.P.; Vaughan, M.M.; Geiser, D.M.; O'Donnell, K., Phylogenetic diversity, trichothecene potential, and pathogenicity within *Fusarium sambucinum* species complex. *PLoS One* **2021**, *16*, e0245037.
8. Yli-Mattila, T.; Ward, T.J.; O'Donnell, K.; Proctor, R.H.; Burkin, A.A.; Kononenko, G.P.; Gavrilova, O.P.; Aoki, T.; McCormick, S.P.; Gagkaeva, T.Y., *Fusarium sibiricum* sp. nov, a novel type A trichothecene-producing *Fusarium* from northern Asia closely related to *F. sporotrichioides* and *F. langsethiae*. *Int. J. Food Microbiol.* **2011**, *147*, 58–68.
9. Knutsen, A.K.; Torp, M.; Holst-Jensen, A., Phylogenetic analyses of the *Fusarium poae*, *Fusarium sporotrichioides* and *Fusarium langsethiae* species complex based on partial sequences of the translation elongation factor-1 alpha gene. *Int. J. Food Microbiol.* **2004**, *95*, 287–295.

10. Son, H.; Seo, Y.S.; Min, K.; Park, A.R.; Lee, J.; Jin, J.M.; Lin, Y.; Cao, P.; Hong, S.Y.; Kim, E.K.; Lee, S.H.; Cho, A.; Lee, S.; Kim, M.G.; Kim, Y.; Kim, J.E.; Kim, J.C.; Choi, G.J.; Yun, S.H.; Lim, J.Y.; Kim, M.; Lee, Y.H.; Choi, Y.D.; Lee, Y.W., A phenome-based functional analysis of transcription factors in the cereal head blight fungus, *Fusarium graminearum*. *PLoS Pathog.* **2011**, *7*, e1002310.

11. Zhang, X.W.; Jia, L.J.; Zhang, Y.; Jiang, G.; Li, X.; Zhang, D.; Tang, W.H., In planta stage-specific fungal gene profiling elucidates the molecular strategies of *Fusarium graminearum* growing inside wheat coleoptiles. *Plant Cell* **2012**, *24*, 5159–5176.

12. Son, H.; Kim, M.G.; Min, K.; Lim, J.Y.; Choi, G.J.; Kim, J.C.; Chae, S.K.; Lee, Y.W., WetA is required for conidiogenesis and conidium maturation in the ascomycete fungus *Fusarium graminearum*. *Eukaryot. Cell* **2014**, *13*, 87–98.

13. Son, H.; Kim, M.G.; Min, K.; Seo, Y.S.; Lim, J.Y.; Choi, G.J.; Kim, J.C.; Chae, S.K.; Lee, Y.W., AbaA regulates conidiogenesis in the ascomycete fungus *Fusarium graminearum*. *PLoS One* **2013**, *8*, e72915.

14. Son, H.; Kim, M.G.; Chae, S.K.; Lee, Y.W., FgFlbD regulates hyphal differentiation required for sexual and asexual reproduction in the ascomycete fungus *Fusarium graminearum*. *J. Microbiol.* **2014**, *52*, 930–939.

15. Fan, G.; Zhang, K.; Zhang, J.; Yang, J.; Yang, X.; Hu, Y.; Huang, J.; Zhu, Y.; Yu, W.; Hu, H.; Wang, B.; Shim, W.; Lu, G.D., The transcription factor FgMed1 is involved in early conidiogenesis and DON biosynthesis in the plant pathogenic fungus *Fusarium graminearum*. *Appl. Microbiol. Biotechnol.* **2019**, *103*, 5851–5865.

16. Lysøe, E.; Pasquali, M.; Breakspear, A.; Kistler, H.C., The transcription factor FgStuAp influences spore development, pathogenicity, and secondary metabolism in *Fusarium graminearum*. *Mol. Plant Microbe Interact.* **2011**, *24*, 54–67.

17. Shin, S.; Park, J.; Yang, L.; Kim, H.; Choi, G.J.; Lee, Y.W.; Kim, J.E.; Son, H., Con7 is a key transcription regulator for conidiogenesis in the plant pathogenic fungus *Fusarium graminearum*. *mSphere* **2024**, *9*, e0081823.

18. Wu, C.; Chen, H.; Yuan, M.; Zhang, M.; Abubakar, Y.S.; Chen, X.; Zhong, H.;

Zheng, W.; Zheng, H.; Zhou, J., FgAP1σ is critical for vegetative growth, conidiation, virulence, and DON biosynthesis in *Fusarium graminearum*. *J. Fungi* **2023**, *9*, 145.

19. Yun, Y.; Liu, Z.; Yin, Y.; Jiang, J.; Chen, Y.; Xu, J.R.; Ma, Z., Functional analysis of the *Fusarium graminearum* phosphatome. *New Phytol.* **2015**, *207*, 119–134.
20. Maeda, K.; Nakajima, Y.; Tanahashi, Y.; Kitou, Y.; Miwa, A.; Kanamaru, K.; Kobayashi, T.; Nishiuchi, T.; Kimura, M., L-Threonine and its analogue added to autoclaved solid medium suppress trichothecene production by *Fusarium graminearum*. *Arch. Microbiol.* **2017**, *199*, 945–952.
21. O'Donnell, K.; Sutton, D.A.; Rinaldi, M.G.; Sarver, B.A.; Balajee, S.A.; Schroers, H.J.; Summerbell, R.C.; Robert, V.A.; Crous, P.W.; Zhang, N.; Aoki, T.; Jung, K.; Park, J.; Lee, Y.H.; Kang, S.; Park, B.; Geiser, D.M., Internet-accessible DNA sequence database for identifying fusaria from human and animal infections. *J. Clin. Microbiol.* **2010**, *48*, 3708–3718.
22. O'Donnell, K.; Kistler, H.C.; Cigelnik, E.; Ploetz, R.C., Multiple evolutionary origins of the fungus causing Panama disease of banana: concordant evidence from nuclear and mitochondrial gene genealogies. *Proc. Natl. Acad. Sci. U. S. A.* **1998**, *95*, 2044–2049.
23. Nakajima, Y.; Akasaka, M.; Shiobara, T.; Kitou, Y.; Maeda, K.; Kanamaru, K.; Ohsato, S.; Kobayashi, T.; Nishiuchi, T.; Kimura, M., Impact of nitrogen metabolism-associated culture pH changes on regulation of *Fusarium* trichothecene biosynthesis: revision of roles of polyamine agmatine and transcription factor AreA. *Curr. Genet.* **2020**, *66*, 1179–1190.
24. Koizumi, Y.; Nakajima, Y.; Tanaka, Y.; Matsui, K.; Sakabe, M.; Maeda, K.; Sato, M.; Koshino, H.; Sato, S.; Kimura, M.; Takahashi-Ando, N., A role in 15-deacetylcalonectrin acetylation in the non-enzymatic cyclization of an earlier bicyclic intermediate in *Fusarium* trichothecene biosynthesis. *Int. J. Mol. Sci.* **2024**, *25*, 4288.
